# Possible Treatment of Myocardial Infarct Based on Tissue Engineering Using a Cellularized Solid Collagen Scaffold Functionalized with Arg-Glyc-Asp (RGD) Peptide

**DOI:** 10.3390/ijms222212563

**Published:** 2021-11-22

**Authors:** Olivier Schussler, Pierre E. Falcoz, Juan C. Chachques, Marco Alifano, Yves Lecarpentier

**Affiliations:** 1 Thoracic Surgery Department, Cochin Hospital, APHP Centre, University of Paris, 75014 Paris, France; alifano.marco@aphp.fr; 2 Department of Thoracic Surgery, University Hospital Strasbourg, 67000 Strasbourg, France; pierreemmanuel.falcoz@chru-strasbourg.fr; 3 Department of Cardiac Surgery, Pompidou Hospital, Laboratory of Biosurgical Research, Carpentier Foundation, University Paris Descartes, 75015 Paris, France; j.chachques-ext@aphp.fr; 4 INSERM U1138 Team «Cancer, Immune Control, and Escape», Cordeliers Research Center, University of Paris, 75014 Paris, France; 5 Centre de Recherche Clinique, Grand Hôpital de l’Est Francilien, 77104 Meaux, France; yves.c.lecarpentier@gmail.com

**Keywords:** RGD, adhesion molecules, contractility, collagen, tissue engineering, myocardial infarct, integrins

## Abstract

Currently, the clinical impact of cell therapy after a myocardial infarction (MI) is limited by low cell engraftment due to low cell retention, cell death in inflammatory and poor angiogenic infarcted areas, secondary migration. Cells interact with their microenvironment through integrin mechanoreceptors that control their survival/apoptosis/differentiation/migration and proliferation. The association of cells with a three-dimensional material may be a way to improve interactions with their integrins, and thus outcomes, especially if preparations are epicardially applied. In this review, we will focus on the rationale for using collagen as a polymer backbone for tissue engineering of a contractile tissue. Contractilities are reported for natural but not synthetic polymers and for naturals only for: collagen/gelatin/decellularized-tissue/fibrin/Matrigel™ and for different material states: hydrogels/gels/solids. To achieve a thick/long-term contractile tissue and for cell transfer, solid porous compliant scaffolds are superior to hydrogels or gels. Classical methods to produce solid scaffolds: electrospinning/freeze-drying/3D-printing/solvent-casting and methods to reinforce and/or maintain scaffold properties by reticulations are reported. We also highlight the possibility of improving integrin interaction between cells and their associated collagen by its functionalizing with the RGD-peptide. Using a contractile patch that can be applied epicardially may be a way of improving ventricular remodeling and limiting secondary cell migration.

## 1. Introduction

Despite a remarkable decline in initial cardiovascular deaths, acute coronary occlusion remains the major cause of heart failure, with a mortality rate of 50% at 5 years [1]. Acute coronary occlusion leads to ischemic cardiomyocyte death and subsequent alteration of contractility due to the impossibility of replacing the loss of cardiomyocytes, at least in a sufficient quantity, given that less than 1% of cardiomyocytes are renewed each year [2]. In addition, myocardial infarct (MI) is associated with a profound alteration of the physical and biological properties of the cardiac extracellular matrix (ECM). MI is associated with the acute degradation of the ECM, which is mostly composed of collagen, its main structural component [3,4]. In rat, 12 h after MI, 75% of collagen type I and type III has already been lost. After 2 days, the collagen progressively increases again with an overcompensation of collagen type I, but not type III at 5 days [5]. In rat, after MI, early left ventricle unloading has been shown to preserve collagen content. However, the original orientation of collagen fibers is still lost. In humans, unloading has been shown to not affect the laminin content of cardiac ECM, but there is a decrease in the very bioactive collagen type IV and an alteration of both the number and quality of the contacts between cardiomyocytes and the ECM [6].

Preclinical and clinical studies have shown that cell therapy attenuates myocardial damage and the progression to heart failure, although the detailed mechanisms involved have not been determined [7,8,9]. After MI, for optimal efficiency, cells should be transplanted as soon as possible, probably within the first 72 h, and possibly in the first few hours [10]. Early stem cell engraftment has been shown to predict late functional cardiac recovery [11]. Today, the clinical impact of cell therapy is limited by low cell engraftment [9]. With respect to the injection of free cells, their impact is limited by a low survival rate, low cell retention, and important secondary cell migration outside the delivery site, and significant cell death by apoptosis in ischemic and damaged inflammatory areas.

Cardiac cells, like most somatic cells, interact with the classic natural ECM through integrin mechanoreceptors that control many cellular signals for cell survival, apoptosis, differentiation, migration and proliferation. Integrins recognize oligopeptides on the proteins of the ECM. Integrins are cell surface receptors that recognize oligopeptides present on proteins of the ECM [12]. For any cells, there are three main types of integrin interactions with the oligo-peptides present on the proteins of the ECM, i.e., collagen, laminin and type “RGD” (Arg-Glyc-Asp) (i.e., oligo-peptide that is present on fibronectin or vitronectin proteins) [12]. For the collagen, the oligo peptide sequences DGEA (Asp-Gly Glu-Ala) and GFOGER (Gly Ph-OHPro-Glu-Arg) are the dominant ligand oligopeptides for integrins [13]. The RGD is present on collagen, but in a cryptic non-functional form for integrins [14]. Gelatin is obtained by heat denaturation of natural collagen and contains a functional RGD that becomes functional during the preparation procedure but, at the same time, the motif DGEA is also lost during the process of gelatin production from collagen. In addition, gelatin polymers have a very low porosity and poor mechanical properties as compared to natural undenatured collagens [15,16]. On 2D and 3D cultures, it has been shown that an early first secretion of laminin by cardiomyocytes on collagen type I, after a few hours, induces and then controls the secondary organization of cardiac sarcomeres [17]. Thus, thanks to this auto-secretion, the laminin signaling is already present in preparations containing collagen and seeded with heart cells [17].

Integrins that recognize the RGD motif have been shown to play a key role during cardiac development [18], pressure overload [19] and after MI [20,21,22] (for review [23]). After infarction, cells involved in the regenerative process are in the epicardial layer [24] and may thus be easier to treat with an epicardial therapy if available. The interaction of regenerative cells with the local RGD motif of fibronectin has been shown to be essential with respect to their functionality and regenerative capacity [21,23]. Animal studies have shown that cellular engraftment is substantially higher if the cells are transferred as a contractile tissue structure rather than via free cell injections or infusions [9]. Three-dimensional cultures have been shown to enhance cell ECM interactions through integrins and intercellular cell–cell interactions [23]. Associating cells with 3D scaffolds is the most promising way to improve cellular transplantation, especially if the cell-containing preparations are applied onto the epicardium instead of being intramyocardially injected [25,26,27,28,29]. Thus, in vitro engineering of a contractile heart tissue, designed to morphologically and functionally resemble the native myocardium before transfer, could enhance cell engraftment, providing a distinct advantage over direct intramyocardial cell injection [9,28,30,31].

Nowadays, the most promising cell types for cell therapy are multipotent human mesenchymal stem cells and resident cardiac stem cell preparations, such as “Cardiospheres” isolated from heart biopsies and embryonic cells: iPS-CM or ES-CM [30]. The crucial role of integrins with regard to therapeutic cardiac cells has been well documented (for review [23]). It has been shown that the present association of human angiogenic progenitor cells, isolated from blood with an injectable collagen type I, enhances their therapeutic effect in the context of an ischemic heart model by enhancing interactions with integrins α5 for fibronectin and α2β1 for collagen on these cells [32]. The attachment, retention and therapeutic benefits of “human cardiospheres” in a SCID mouse model of infarct have been shown to be dependent on the pre-transplant β3 integrin expression on these cells that later determines the attachment of the cells to the fibronectin of the MI area [33]. Up to now, the functionality of all these preparations containing cells with contractile capability has been shown to be related to the paracrine potential of the associated cells and not their effective contractility [30]. On the other hand, contractile patches, unlike non-contractile patches, may enhance external myocardium remodeling. In addition, there is some evidence that contractile cells do have lower migration capabilities than non-contractile cells [34], and that may help for their therapeutic retention in the specific areas to be treated.

In vitro, contractilities have been demonstrated with hydrogels, gels and solid scaffolds [35,36]. With respect to tissue sheet technology, thicker patches have been shown to necessitate the incorporation of gelatin hydrogels between layers, while long-term contractility in vitro is still a challenge [37]. Up to now, contractilities have been reported only with preparations containing natural polymers such as collagen itself, gelatin (i.e., that is the heat-denatured form of natural collagen) or decellularized tissues that are mostly composed of collagen. Contractilities have also been observed in preparations containing natural polymers such as fibrin [38], but not with synthetic materials alone or with biological materials such as alginate [39] or chitosan [28,40]. Contractilities have also been reported with Matrigel™ [41,42,43,44] (that are composed of proteins and growth factors isolated from a tumor extract of basement membrane proteins) and thus cannot be used in humans.

For long-term contractility in vitro, angiogenesis and the tissue engineering of a thick contractile construct and in vivo transfer, solid scaffolds are superior to hydrogel or gels [35,36]. In this review, we emphasize the rationale for having a solid scaffold for engineering a contractile tissue in vitro and for cellular therapy. The review provides details of the main methods for obtaining a solid 3D scaffold (for reviews [13,15]), such as physical reticulation dehydrothermal treatment (DHT), electrospinning, bioprinting or solvent casting, and describes the classical methods for reticulation and the main observations after seeding contractile cells.

Our research has been the first to demonstrate, firstly in mice [45] and then later in humans requiring a bypass after MI [46,47], how the epicardial application of a solid collagen patch, obtained by DHT and already in clinical use as a local hemostat and cellularized with patient autologous bone marrow cells, is superior to the classical intramyocardial free-cell injection.

The interest of having a collagen scaffold functionalized with RGD in the context of MI or for the engineering of a contractile tissue has never been reviewed. In highly porous and highly compliant solid collagen scaffolds obtained by dehydrothermal treatment (DHT), very interesting long-term and thick tissues have been achieved with neonatal rat cardiomycoytes, but the tumor extract of basement membranes (i.e., Matrigel ™) has been found to be necessary for cell seeding and survival [41,42,43]. However, the presence of this gel altered nutrient diffusion so that a bioreactor is also needed [41,42,43]. The “RGD” (Arg-Glyc-Asp) is present on natural undenaturated collagen, but is not functional and thus cannot interact with integrins [14]. By using neonatal rat cardiomyocytes in a porous, highly compliant solid collagen scaffold, obtained by physical reticulation through dehydrothermal treatment (DHT), we have demonstrated how its functionality could be improved after functionalization with the RGD through a new method for ligand binding and presentation [48]. Then, it has been possible to engineer a very efficient contractile tissue, in vitro and for a very long period of time (more than 1 month), without the need for Matrigel™ and bioreactors for perfusion [48], as reported earlier by other research groups [41,42,43,49,50]. More recently, we have also reported in vitro how the solid collagen RGD scaffolds increase the cardiogenic potential of clinical “Human Cardiospheres” compared with gelatin-based solid foams of the same stiffness or the classical cultures such as “3D cell clusters” [40]. We have also recently demonstrated in vitro how the presence of RGD-functionalized collagen promotes the differentiation of human mesenchymal stem cells (MSCs) towards a contractile phenotype of myofibroblasts and improves contractility by enhancing actin-myosin crossbridges [51,52,53]. In a 3D construct of collagen, we have also demonstrated that the fundamental MSC paracrine activity is preserved, even after differentiation into contractile myofibroblasts. The paracrine activity in 3D scaffolds has even been shown to be superior to that in classical 2D cultures [51]. This observation has been confirmed by another research group, but in their experiments, the MSCs in collagen scaffolds are still associated with Matrigel™ [54]. The RGD peptide is also known to enhance angiogenesis in the presence of endothelial cell progenitors [23] and thus may also facilitate construct survival and functionality upon implantation. We anticipate that the functionalization of the collagen polymers used for making 3D environments for epicardial or intramyocardial cell delivery may well be improved with the RGD peptide that is present, but not functional, on native collagen [29,48].

## 2. Different Approaches for Cardiac Cell Delivery after MI

After MI, the main cellular effects after cell transfers are not due to the true contractile potential of the preparations, but more to the paracrine capabilities of associated cells, whether they are contractile or not. In general, a tissue engineering or regenerative medicine approach consists of seeding cells in a scaffold, followed by in vitro tissue maturation and construct implantation in the host environment. However, alternative approaches exist, but lack some elements or steps such as (i) cell injection with or without a scaffold (no in vitro maturation) and (ii) scaffolds that attract endogenous cells (no cells and in vitro maturation). All these approaches involve the design of a pre-formed or injectable scaffold, using a biomaterial able to properly interact with seeded or endogenous recruited cells. Therefore, surface functionalization can be employed in both seeded and unseeded scaffolds.

### 2.1. Use of Cells Associated with a 3D Scaffold for Tissue Engineering and Cell Transfer

An ideal 3D scaffold should provide: (1) a biomimetic diversity of binding sites to engage functionally relevant integrins in both cardiomyocytes and non-myocytes; (2) a sufficient numbers of cell-binding sites to permit a physiological tissue density; (3) a capacity for significant remodeling of tissue structure to allow rapid cell spreading, alignment and replacement of original scaffold with cell-secreted ECM; (4) an appropriate biomechanical properties to enable continuous tissue contraction; (5) a minimal immunogenicity when transplanted in vivo; and (6) a stability to allow generation and implantation of tissues of clinically relevant size.

The development of suitable biodegradable biomaterials as candidates for cardiac tissue engineering is an active field of research. Different methods are continuously studied to develop three-dimensional scaffolds with a specific shape, thickness, mechanical strength, and porosity to promote cell growth. The specific physical properties of constructs that are crucial for the success of this approach are biocompatibility, the chemical composition of the polymers, possibility of absorbing proteins, surface energy, adhesion molecules, ability to foster cells, tailored degradation rate, permeability (for biomolecule diffusion), natural or non-natural components, adhesion molecules, porosity, ability to absorb proteins, suitable mechanical properties (stiffness [55], viscoelasticity [56,57]), ultrastructural properties (orientation, roughness, fibrillary network) [58], nanotopography [59], contractility and electrophysiological stability, toxicity degradation, low immunogenicity, ability to promote angiogenesis, ability to engineer a thick scaffold of sufficient size, clinical safety, and stability (for recent reviews [26,28,60]).

**Figure 1:**
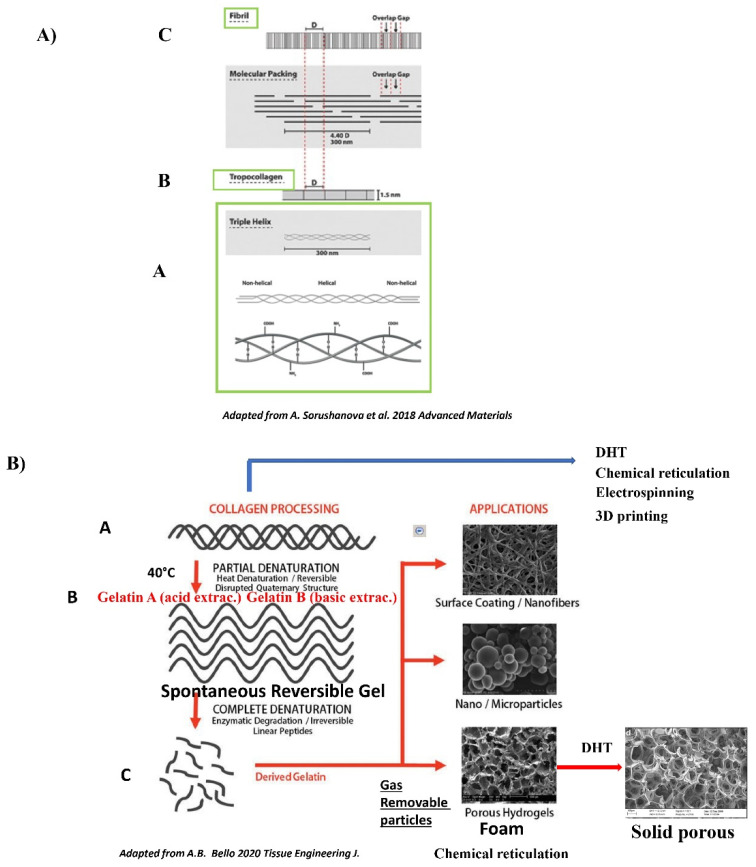
Collagen and gelatin-based polymers. (**A**) Collagen structure and organization. The collagen primary structure contains the RGD sequence, but is inside the triple helix and therefore not functional. Panel A: The collagen monomer is organized in two helical regions and two non-helical regions. The collagen triple helix (tertiary structure) has a coiled-coil structure made of three parallel polypeptide α chains (secondary structure). Panel B: The collagen monomers will spontaneously self-assemble end to end to form tropocollagen and also laterally to form a larger structure, such as fibrils. Panel C: Schematic representation of the arrangement of collagen molecules within fibrils (*adapted from A. Sorushanova* et al. *Adv. Mater. 2018* [16]). (**B**) Due to its triple helix and spontaneous propensity to self-assemble into more complex structures, the collagen has exceptional properties in terms of resistance to temperature, its ability to form a highly interconnected porous network with a specific microarchitecture, and its exceptional mechanical resistance to stress and degradation. The collagen can be further processed to form more elaborate 3D constructions of hydrogel, gel or solid constructs using DHT processing, electrospinning or bio-printing. The different approaches can be combined and the different supports can be cross-linked physically, chemically or enzymatically. In some applications, the collagen can be denatured by heating to over 40 °C to form gelatin (panel B). In gelatin, the quaternary structure of collagen is lost as well the collagen triple helix. Gelatin is highly soluble and forms a spontaneous but reversible gel when temperature decreases depending on concentration and pH level. When the temperature falls below 30 °C, a reversible coil of triple helix occurs and hydrogen bonds stabilize the conformation. This physical gelation method is reversible and the gel needs to be cross-linked. The collagen can be further processed using enzymatic degradation that will lead to small linear peptides of collagen with irreversible degradation of the triple helix (panel C). The gelatin can be used to form more semi-porous foam by means of removable gas or particles. Gelatin-based scaffolds have a lower porosity than the equivalent obtained with undenaturated collagen monomers. In gelatin, the RGD binding site becomes functional, but other important adhesion sites such as the GxOGER binding site are lost during the transformation (*Figure adapted from Bello A.B.* et al. *Tissue Eng.* [15]).

Biomaterials are designed to mimic the intricate native cardiac ECM, mainly composed of collagen. Common methods include the control of the mechanical properties of the material, incorporative bioactive signals, spatially patterning bioactive signals and the controlled release of bioactive signals. Both natural polymers (collagen monomers, gelatin), fibrin glue (fibrinogen), natural polysaccharides, such as alginate/chitosan/hyaluronic acid, and synthetic polymers have been used. The best results so far have been achieved with natural polymers. Natural materials such as collagen (Figure 1), fibrin or synthetic polyglycolic acid have been widely investigated, and some novel compositions (i.e., silk fibroin and hyaluronic acid, alginate/chitosan polyelectrolyte complexes) have recently been introduced.

Despite the fact that they show the required biocompatible behavior, most synthetic polymers exhibit poor cell attachment capability. These synthetic polymers are mostly hydrophobic and lack cell adhesion recognition sites, limiting their applications. Biofunctionalization of these materials with oligopeptides recognized by cell surface integrin receptors and recapitulating most of the full protein biofunctionality have been widely investigated [56,57,61,62,63].

Cells, including cardiac heart cells (i.e., cardiomyocytes [64], fibroblasts/myofibroblasts [58], endothelial cells [65]), are recognized by their cell surface integrin mechanoreceptors, short oligopeptide sequences present on proteins of the ECM. These short peptide sequences recognized by integrins on proteins are for the collagen (i.e., the “GFOGER” oligopeptide), for the laminin (i.e., “IKVAV” or “YIGSR “oligopeptides) and for the fibronectin or vitronectin (i.e., the “RGD” peptides) [62]. Integrins control many cellular processes such as cell survival, death, apoptosis, migration, differentiation and proliferation (for recent reviews [29,59,60,61,62,63,64,65]). The fibronectin [66], especially its portion containing the RGD fragment and laminin, has been shown to play a key role during cardiac development. Integrins recognizing the RGD peptide have also been shown to play a key role after MI (For review [3,4]).

Natural polymers composed of polysaccharides (such as alginate/chitosan/hyaluronic acid) also lack adhesion molecules and have a low propensity for spontaneous protein adsorption. Various materials have been tested, including 3D “gel” and solid 3D porous sponges made of alginate, collagen or gelatin, polyglycolic acid, poly-l-lactic acid/polyglycolic acid composites, and poly(glycerol sebacate). An obvious advantage of solid scaffolds when compared with gels is the ease of engineering any desired 3D form and thickness for a prolonged period of time. The solid scaffold may also facilitate cell transfer. Complex micro tissues have also been obtained in synthetic poly(glycerol sebacate) seeded with cardiac fibroblasts and neonatal cardiomyocytes. Beating cardiomyocytes have been reported, but not a true contractility of the structure with force and displacement has not been clearly demonstrated [66].

Recent advances in nanomaterial technology have driven the design of more complex microenvironments mimicking those of native myocardium. Specifically, sophisticated methods for electrospinning (Figure 3, panel II) and 3D bioprinting (Figure 3, panel III) offer the ability to use natural and/or synthetic biomaterials and to control scaffold architecture and cellular composition in a spatially precise fashion [26]. Electrospinning involves extrusion of electrically charged polymers through a small orifice to create nanofibers with precisely controlled dimensions and the ability to generate higher-order 3D geometries based on fiber collection methodologies (Figure 3, panel II). No contractility has been demonstrated even in composite scaffolds. Most electrospinning studies have utilized synthetic polymers (PLGA, polycaprolactone (PCL). Recent methodological advances have permitted increased incorporation of native proteins (e.g., gelatin, collagen) into electrospun nanofibers. There are several limitations that must be overcome, however, to enable the generation of highly functional and therapeutically relevant contractile tissue using the electrospinning approach. These include: (1) low porosity that precludes deep penetration of seeded cells; (2) inability to efficiently incorporate various cardiac ECM proteins; (3) very stiff constructs (around 1000 kPa), while the optimal stiffness for cardiac tissue engineering is around 10–20 kPa; (4) the ultrastructure of the collagen fibers that are lost; and (5) very small size pores (around 1–3 μm), limiting cellular colonization. The new 3D bioprinting technology allows the fabrication of contractile tissues even with the association of natural and synthetic biomaterial polymers (Figure 3, panel III). An early work has utilized the RGD-conjugated alginate or HA/alginate matrices with human cardiac progenitor cells (hCPCs) and 3D-print. The 3D printing technology allows applicability in the fields of tissue engineering and regenerative medicine in the form of bioprinting. Bioprinting involves the process of laying down cells in a predefined spatial arrangement with or without use of a biocompatible scaffold, using 3D printing technology. In order to obtain a functional tissue, the cells must maintain their viability and specific cell function within their new environment. Three-dimensional bioprinting strategies have been shown to be valid in creating a functional cardiac tissue capable of synchronized contractions with the characteristics of native myocardium, although tissue thickness is still very low. After 3 weeks of culture with neonatal rat cardiomyocytes in 3D bioprint hydrogels of fibrin/hyaluronic acid and gelatin, a developed force of 2 mN has been reported. The entire construct is 1.8 × 1.6 cm^2^ with a 0.6 mm thickness [67]. More recently, an oriented cardiac microtissue with neonatal rat cardiomyocytes has been made by 3D bioprinting with polyethylene glycerol, polydimethylsiloxane (PMDS), and gelatin methacrylate. Scaffold stiffness is high, around 148 kPa. A thin construct of less than 100 μm was fabricated and a small displacement of around 10μm has been observed with a maximum developed force of 14 mN/mn^2^ [68].

### 2.2. Possible Transfer of Cells in a 3D Construct without a 3D Scaffold

#### 2.2.1. Culture of Cells In Vitro as a 3D Cell Cluster: The “Spheroid Approach”

The development of cardiac cells as 3D clusters has been shown to be an efficient method for cardiac cell isolation, amplification and transfer [69,70,71]. This approach has been used for human “cardiosphere” isolation and amplification and to enhance cell paracrine functionality. The culture of cardiac cells as a 3D cluster is a way for enhancing the cell–cell interaction. A neo ECM has also been observed. The major drawback is the lack of functional and homogenous architecture, meaning that there is no construct true contractility [72]. Achieving an initial uniform distribution of the cluster at the time of cell delivery is also not a trivial task. In some cases, the association of spheroid cells with 3D scaffolds can improve their transfer to intra myocardial or epicardial locations [73].

#### 2.2.2. Cell Sheet Technology

Cardiac myocytes cultured on standard plastic dishes for extended periods tend to detach from the substrate as a more or less intact monolayer. For a recent review on the topic for tissue engineering a contractile tissue with tissue sheets [74]. Several monolayers can be overlaid to form 3D structures. Shimizu et al. have exploited this principle in developing temperature-sensitive coating materials that allow cell monolayers to detach, intact, from the culture surface at room temperature [74]. Stacking of several cell sheets generates 3D tissues that beat and develop force. One limitation of this approach is the diffusion, which is limited to a few cell sheets (total thickness of about 80 μm) and poor angiogenesis. With this approach, using human iPSC-derived cardiac cells in fibrin sheets, the contractile force has been reported to be 0.85 mN and the thickness 21.5 μm [74]. More recently, thicker viable cardiac tissue up to 1 mm has been obtained in vitro by tissue overlays after incorporation of gelatin hydrogels between layers, endothelial cells and also the use of bioreactors and system of preformed extrinsic vasculature with collagen [37]. Using this approach, shortening has been reported, but with no force [75]. In vivo, the contractile preparations after an epicardial application have been shown to survive and become electrically connected with functional improvement after MI [37].

## 3. Different Three-Dimensional Materials for Tissue Engineering and Contractilities Observed after Seeding with Cells with Contractile Potential

Contractility has been reported after seeding contractiles cells in hydrogel, gel or soft solid three-dimensional materials.

### 3.1. Different States of Three-Dimensional Materials: Hydrogel, Gel or Solid for Tissue Engineering Contractile Tissues

**Figure 2:**
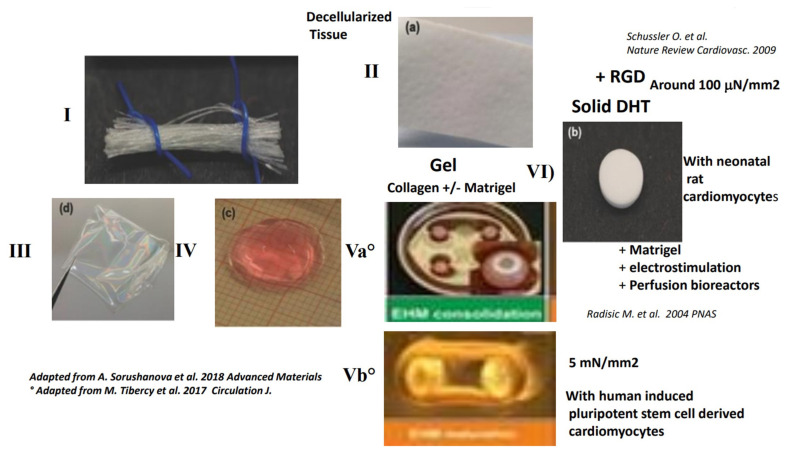
Several forms of collagen used in biomedicine to engineer a contractile tissue after seeding with contractile cells. Adapted from A. Sorushanova et al. Advanced Materials 2018 [16] and from M. Tiburcy et al. 2017 Circulation J. [79].

The three-dimensional material can be hydrogel [76], gel [77] or solid. With respect to the 3D material containing the collagen as a backbone, it may be a hydrogel of collagen, a solid gel of collagen, a solid scaffold of collagen, or a tissue-decellularized path that also has a very high collagen content, since the collagen is the predominant component of the ECM (for review [13,16,78]) (Figure 2).

There are two main ways of using collagen. One approach consists of extracting the collagen from the tissue as fibers (Panel I), while the other uses the decelularization procedure to obtain a decellularized tissue that is known to contain a large amount of collagen and then to directly seed the cells in the decellularized tissue (Panel II). Contractility is observed after seeding contractile cells in decellularized tissue or on a thin cross-section of the decellularized tissues.

Diluted acid solutions with or without enzymes, neutral salts and alkali treatments are used to isolate the collagen from the different tissues. Such solutions effectively dissociate intermolecular aldimine crosslinks (between triple helices), but they are ineffective against more stable and mature crosslinks. In the latter case, proteolytic enzymes, especially pepsin, are employed. The cells can be cultured onto a 2D film of collagen (Panel III) and then applied to the myocardium. This approach facilitates cell delivery and implantation. However, the preparations are not thick enough to have an efficient contractile tissue even when several overlying sheets are used.

With a neutral physiological pH, the collagen will spontaneously form a hydrogel (Panel IV) or gel (Panel V) depending on ionic strength and temperature following an entropy-driven process. The association of cells (i.e., human embryonic cells) with a gel of collagen and Matrigel™ has been used to develop one of the most effective contractile preparations in vitro, especially if prolonged physical stimulation is applied (Panel Vb) [79]. Very promising results from using a thick patch (around 3 mm) and long-term constructs have also been achieved by seeding contractile cells (i.e., neonatal cart cardiomyocytes) in a 3D solid, highly porous and very supple collagen scaffolds obtained by DHT in the presence of Matrigel™ (Panel VI) [41,42,43]. In this setting, we have shown that it is possible to replace the Matrigel™ by the functionalization of the solid collagen scaffold with the RGD (Panel VI) [48].

For the collagen polymer, it can be the native nature collagen or its heat-denatured derived form (i.e., gelatin) [16,80]. Three-dimensional solid structures (i.e., known as compressed hydrogels or gels), obtained by physical compression of hydrogels and gels, have also been reported [81].

Hydrogels [76] are efficient three-dimensional materials for tissue engineering thanks to their high water content and flexibility, which allow them to mimic native ECM. Hydrogels are formed when a three-dimensional polymeric network is loosely crosslinked. They are swollen by water, but not dissolved in it. Hydrogels can display reversible sol–gel transitions, induced by changes in the environmental conditions such as temperature, pH, ionic strength, phase separation, wave length of light, and crystallinity. Hydrogels are described as “smart or intelligent” when a sharp transition is induced by a small changes in such conditions. For the shape-memory hydrogels, a reversible change in shape can also be induced by such stimuli [82]. Hydrogels have the ability to maintain large amounts of water with tunable biocompatibility, biodegradability, acute environmental sensing, and mechanical properties. They can be designed by the incorporation of natural or synthetic polymers through physical or covalent cross-linking and can be divided into two categories based on the molecule types formed: natural polymer and synthetic polymer. Commonly, naturally derived hydrogels including cellulose, chitosan, alginate, and agarose are common in the natural environment. They can be temperature- or light-sensitive or have shape-memory properties [76,83]. Hydrogels can also be injectable [76,84], using polymeric biomaterials that undergo a solution to gel phase transition, and may incorporate embedded cells and/or active compounds. Adaptable hydrogels have been used with hyaluronic acid and for targeted therapeutic delivery (i.e., miR) to the heart in mice [85].

A gel is a solid comprising at least two components, one of which (the polymer) forms a three-dimensional network by virtue of covalent or noncovalent bonding (chemical and physical gels, respectively) in the medium of the other component (liquid), wherein the minimum amount of liquid is sufficient for ensuring the elastic properties of the gel, although it can exceed, by many times (tens to hundreds), the amount of the polymer [86]. Gels can be embedded with cells and injected under pressure into tissues with or without cells incorporated. It should be noted that, with a high network density or high polymer-chain rigidity, the formation of fragile gels is possible. A general feature of physical gels is the existence of the yield point [44]. The structural properties of gel can be improved by reticulation (physical, chemical or enzymatic). Cross-linking within the gel’s polymer or colloidal network causes a gel to behave as a solid in its steady-state and makes it feel tacky. However, most of the mass of a gel is liquid, so gels can flow following the application of relatively little pressure. Collagen gels are flowable, suggesting the possibility of an easily injectable, biocompatible drug delivery matrix. Fibrillar collagen gels have an effective pore size of several tens of nanometers, too large to control their release by hindered diffusion. To control release, it is necessary to rely on the binding of the active agent to collagen, either by covalent or non-covalent bonds, or on sequestering in a secondary matrix. Such steps rapidly increase the complexity of the system. Non-fibrillar collagen has a lower effective pore size (4–6 nm), but it dissolves rapidly in vivo (approximately 24 h). For tissue engineering applications, collagen gels are more attractive, since they can act as a “cage” to retain cells or as gene delivery complexes. The gels have limitations in terms of strength, but reinforcement with solid components and alignment during gelation and culture can improve performance [44].

Patches or scaffolds are solid polymeric matrices that are highly porous with interconnected polymer networks hydrated with a liquid allowing cell attachment and proliferation and the transport of nutrients and metabolic waste. Generally scaffolds are seeded with cells or made to allow native cell infiltration if implanted [13]. The different polymers can be functionalized with biomolecules [63,87]. As with gels or hydrogels, the scaffold can be additionally cross-linked to increase its stiffness, control its biodegradability and maintain its architecture.

### 3.2. Composition and Key Parameters of the Three-Dimensional Materials for Engineering a Contractile Tissue: Polymer Composition, Adhesion Binding Sites, Porosity, Alignment, Architecture, Microarchitecture, Stiffness, Viscoelasticity 

For review [35,36].

Scaffolds frequently used in tissue engineering have compositions that mimic the composition of the native ECM of soft tissues (i.e., containing both collagen and elastin). Collagen typically gives the tissue its mechanical strength and stiffness, while elastin can provide elasticity and the ability to store elastic-strain energy. Collagen’s relatively high tensile strength and stiffness (120 MPa and 1.2 GPa, respectively), when hydrated, means that even small changes in its concentration, type, crosslinking and spatial alignment in the ECM can lead to significant effects on the mechanical properties of the tissue. Heart muscle has been shown to contain approximately 75%–90% collagen and up to 25% elastin (dry weight), where the collagen type I represents 60%–85% and collagen type III 15%–40% [3,19]. It should be noted, however, that each tissue has its own set and content of proteins and biomolecules, so that attention should be paid to combining the appropriate proteins to provide the optimal physical properties and microenvironment for cells. Various different materials have been used to produce scaffolds for cardiac tissue engineering associating natural polymers (such as collagens associated or not with glycosaminoglycans or gelatin (such as Gelfoam™)) with synthetic polymers [13,16]. However, little consideration has been given to how the composition or crosslinking may affect the physical properties of the scaffold. The impairment of cardiac function with disease is associated with a change in the ECM composition, and more specifically an increase in global collagen content, a change in collagen proportions (an increase in less compliant collagen type I and a decrease in compliant collagen type III), increased crosslinking and a decreased amount of elastin [16].

Varying the protein composition of scaffolds can provide a means of tailoring their mechanical and degradation properties. The thermal denaturation may alter the physical properties of the material as well as the available cell binding sites. It has also been suggested that an enhanced collagen crosslinking can lead to a stiffening of soft tissues. Crosslinking using carbodiimides, such as 1-ethyl-3-(3-dimethylaminopropyl) carbodiimide hydrochloride (EDC), is via carboxylic acid and amine groups on the proteins (both collagen and elastin) and has been shown to increase their protection against degradation and their mechanical properties [13]. In the production of crosslinked scaffolds, the degree of crosslinking should be sufficient to provide resistance to degradation, but not to exceed the mechanical properties of the native myocardium [88].

It is now well established for 2D and 3D environments that integrins and stiffness are directly associated with cell behavior. Matrix stiffness has been shown to control mesenchymal stem cell key behavioral characteristics, such as adhesion, migration, differentiation and cell death [88]. As with 2D environments, a certain stiffness (around 8–12 kPa of MSC) has been shown to be crucial to stem cell differentiation in 3D scaffolds towards contractile cells [57]. In 2D and 3D environments, it has recently been shown that the density of adhesion peptide, stiffness and viscoelasticity determine MSC differentiation capability [57]. With human embryonic totipotent stem cells, it has also been shown recently that a compliance of around 12 kPa is optimal for their differentiation towards cardiomyocytes and the organization of their contractile sarcomere [89].

Oriented surface 2D and 3D environments have been shown to promote differentiation towards contractile cells [90] and to increase intercellular coupling (i.e., electrical and mechanical coupling). In 3D environments, orientation can be obtained during the construction of the 3D scaffold through electrospinning, DHT or bioprinting, or by using mechanical forces during collagen gel alignment or a flow of collagen though microfluidic channels during fabrication [78].

### 3.3. Common Methods Used to Fabricate Solid 3D Scaffolds including Those Used for Contractility

**Figure 3:**
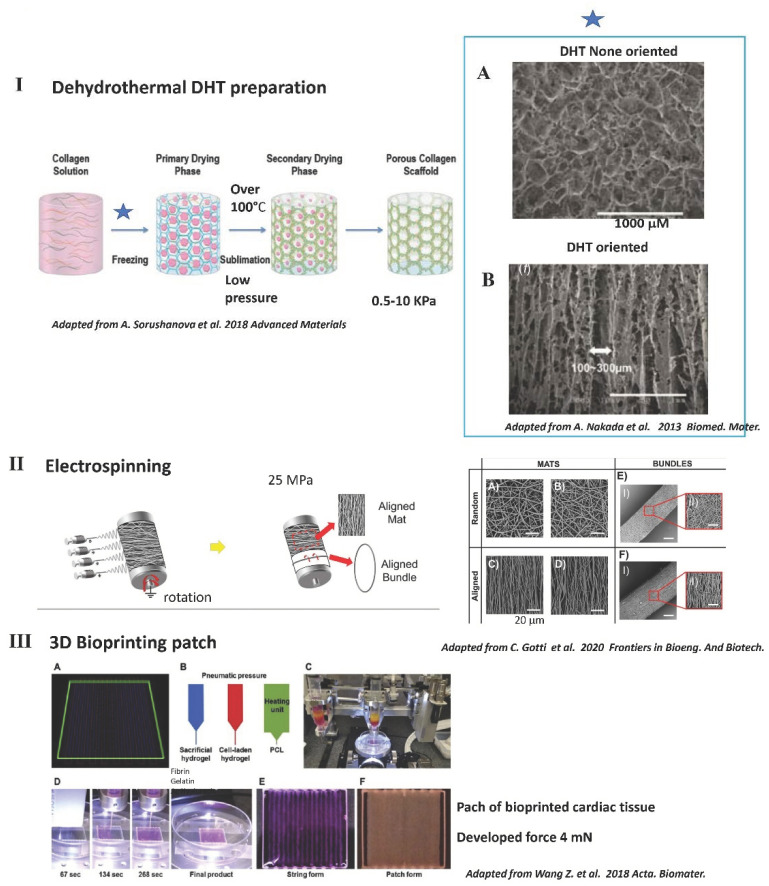
Classical methods for obtaining a 3D solid scaffold with collagen. The most commonly used methods are dehydrothermal treatment (DHT) (panel **I**), electrospinning (panel **II**) and 3D bioprinting (panel **III**). The DHT procedure comprises two phases. During the first phase (i.e., freezing), the temperature is below 0 °C and the gradient determines the size of the water ice crystals that in turn determine the porosity of the scaffold. It is also possible during this first phase to create a temperature gradient to obtain an oriented scaffold, as reported (panel A and B) by Nakada et al. [93]. During the second phase of the procedure, the immediate application of a high temperature (above 100 °C) and a low pressure will provoke a sublimation of the ice crystals and the elimination and dehydration of the collagen structure, leading to local physical crosslinking. The DHT scaffolds have a very low stiffness value (between 0.5–10 kPa). Their high porosity (30–200 μM) for nutriment diffusion, the microarchitecture of collagen and their viscoelasticity have been shown to be very effective in achieving some of the most promising possibilities for long-term contractile scaffolds in vitro. Electrospinning is another interesting approach to obtain an oriented 3D scaffold (Panel II). The porosity of the scaffolds is very low (around several μm), the stiffness is still very high (around 25 MPa) and the collagen microarchitecture is lost. In addition, the solvent used for electrospinning alters the collagen structure. To date, no contractility has been reported when using this approach. Finally, 3D bioprinting (Panel III) is a very promising strategy to obtain a solid 3D scaffold for engineering contractile tissue. Patches made of gelatin/hyaluronic acid or fibrin with hyaluronic acid, all crosslinked with a thiol linker have been developed in the presence of human embryonic stem cells and interesting forces around 2 mN have been reported. However, one limitation of the approach is the very limited thickness of the preparation around 0.6 mm, while the myocardium is around 10 mm (*adapted from Wang Z.* et al. *Acta. Biomater. 2018* [67]).

Common methods used to obtain solid scaffolds are: (1) electrospinning, (2) freeze-drying (DHT), (3) 3D printing and (4) solvent casting (Figure 3) [91,92].

(1) Electrospinning (Figure 3, panel II) consists of stretching the polymer solution or melting it into a fine stream by an electrical force and then solidifying it to fabricate continuous and uniform fibers from nanoscale to microscale (for review [78,94]). By adjusting the spinning parameters, electrospinning can be used to obtain fibrous oriented scaffold materials, but still with a low porosity and high stiffness. Current electrospinning methods use the solubilization of collagen in a solution that also alters the collagen. In addition, the pore size is very small (2 μm) and not ideal for cellular colonization. Scaffolds obtained by electrospinning are very rigid (above 100 kPa) and thus not ideal for engineering a contractile tissue [94]. On the surface of electrospun scaffolds, synchronized beating cells have been demonstrated, but no true contractility has been observed following the development of force or the reversible shortening of the preparation (Figure 3, panel II) [95].

(2) Freeze-drying (Figure 3, panel I), also known as “lyophilisation”, is used to sublimate the solvent in frozen materials at a relatively low pressure in order to dry them (Figure 3, panel I) [96]. The scaffolds are able to keep the original shape and primary structure, and also demonstrate excellent quality after rehydrating. Freeze-drying has been used to improve the porosity and mechanical properties of gel and hydrogels [97]. However, the physical parameters of freeze gel or hydrogel are less homogenous and the quality is far below that that obtained with a solid scaffold produced directly by DHT without using an intermediate gel or hydrogel during manufacturing. During the DHT process, there is also some alteration of collagen fibers, and some preparations need further chemical reticulation depending on the application [16].

(3) Three-dimensional bioprinting (Figure 3, panel III) is a new method used to build the three-dimensional stereoscopic structure by superimposing printed materials, typically layer by layer. Owing to its controllability, repeatability, and precision, 3D printing shows promising applications in the preparation of scaffolds with complicated structures. In addition to optimizing 3D printing techniques, it is also vital to select materials with good rheological properties, similar to those of natural ECM, and biocompatibility for skin scaffolds, among which NFC would appear to be a suitable candidate. To further improve stability and fidelity, treatments such as cross-linking and incorporation with other auxiliary materials are preferable for NFC-based scaffolds. True contractile tissues have been developed recently in vitro using a bioprinting technology with neonatal rat cardiomyocytes [67,68].

(4) Solvent casting often obtains membranes, which involves three steps, dissolving polymer in solvent, casting the solution into a mold, and evaporating the solvent. Tissues have been developed with nanocellulose but the tissues are very rigid and no contractility has been demonstrated so far [91].

## 4. Contractility Reported for Different 3D Materials (Depending on Polymer Composition) or with Decellularized Tissue

Besides the tissue sheet approach, to date, contractility has only been reported after seeding cells in preparations containing collagen (such as natural collagen, gelatin or decellularized tissues), fibrin or Matrigel™ and sometimes associated with synthetic polymers. No contractility has been reported after seeding contractile cells in natural alginate or synthetic materials alone. Contractility has been reported with preparations of hydrogels, gels or solids.

Contractile cardiac tissues can be constructed: (1) by seeding the cells onto ECM of decellularized myocardial tissue; or (2) by seeding cells in a 3D scaffold. Mechanical forces have been shown to guide cardiogenesis [98] and to control contractile cardiac development [99]. It has been shown that engineered contractile tissue could be transplanted into the heart surface epicardium. At this location, preparations survive and become vascularized and even integrated functionally, electrically and mechanically. Thus far, contractility has only been reported for natural scaffolds containing collagen (i.e., collagen, gelatin (i.e., denatured collagen, but with very poor structural properties) [100,101], decellularized tissue that also mainly contains collagen) or with natural fibrin [102]. No contractility has been demonstrated with natural polymers, such as polysaccharide (alginate, chitosan, hyaluronic acid), or with synthetic polymers, such as PGA, PLA, PLC and polyurethanes poly(glycerol sebacate) (PGS), even if they are associated with collagen [103]. The self-assembling peptide technology with “RAD” has failed to demonstrate the capability of obtaining a preparation with contractile properties in vitro and in vivo. Using this same technology (RAD), neonatal rat cardiomyocytes also have a low survival rate following implantation [104].

Contractility has been initially evaluated with animal neonatal rat cardiomyocytes before the recent development of cardiomyocytes derived from human embryonic cells. Again, as in earlier experiments with animal cells, optimal contractility in vitro and in vivo has been obtained for preparations containing several cell types: 65% cardiomyocytes, 25% fibroblast, and 5% endothelial cells. Most methods (i.e., electrical stimulation, mechanical stimulation, bioreactors and 3D scaffolds (polymers used or a combination of polymers), method of production, orientation, nanotopography [59]) developed with neonatal rat cardiomyocytes [105] have also been shown to work for human-derived cardiomyocytes in promoting their survival and maturation. The contractile tissues with human cells were also shown to have engraftment following heart epicardial implantation [28,79,106,107,108,109]. In vitro, cells require a 3D environment with a stiffness of around 10kPa to achieve terminal maturation of the contractile apparatus [98,99]. More recently, it has been shown that beside stiffness [55], viscoelasticity also controls the differential potential of stem cells [56]. Collagen microarchitecture, such as the presence of a microfibrillar architecture, has been shown to be an independent factor in promoting the differentiation of contractile cells [58]. While the collagen as a natural structural protein has a natural viscoelastic component, this is not the case for most other polymers. The collagen, as a natural polymer, is the main component that determines the viscoelasticity of the heart myocardium [56]. Crosslinking of the 3D scaffold is associated with a decrease in this viscoelastic component [56].

In an emulsion in which the polymer and cells are not in contact, no contractility has ever been demonstrated. For long-range force transmission in physically connected polymers, such fibrillar hydrogel or lyophilized “sponges” will help the gel to contract even in the absence of local cell–cell contractility. The scaffold will also help for cell migration, orientation, survival, differentiation and proliferation. In physically cross-linked collagen or reversibly cross-linked alginate gels, the matrix will undergo local plastic deformation, so that forces are dissipated throughout the matrix. The force transmission to neighboring cells decreases more rapidly as a function of distance from the contracting cell compared to a more elastic (less plastic) material. These basic concepts in contractility and material properties may explain why certain materials, such as DHT or EDC-crosslinked collagen, might perform better than collagen alone. The reticulation is also a way to control and increase scaffold stiffness to an optimum level if needed and to maintain architecture and physical properties for a prolonged period of time.

### 4.1. No Contractility in 3D Scaffolds Made of Polysaccharides Such as Alginate or Chitosan, Even with an RGD Peptide

Polysaccharides are molecules that display high biocompatibility and biodegradability. Most natural polysaccharides present groups such as hydroxyl, carboxyl and amino groups, which make their chemical modifications quite easy. Alginate is one of the most studied natural polymers investigated for cardiac tissue engineering, but no contractility has been demonstrated for it so far [39]. Alginates have two main limitations: the absence of adhesion molecules and the low capability for spontaneous protein absorption [39]. The use of alginates in association with collagen as composite microbeads encapsulating neonatal rat cardiomyocyte cells has been developed recently [39]. The composite promotes the proliferation of cardiac cells, the formation of interconnected multilayer heart-like tissues, the presence of well-organized and dense cell structures, and spontaneous synchronized beating, but again there has been no true contractility demonstrated [110,111].

#### 4.1.1. No Contractility in Soft Hydrogel of Alginate, Even with the RGD Peptide

For review [39].

In vitro, the 2D culture of neonatal rat cardiomyocytes on alginate hydrogel, even functionalized with the RGD peptide, has failed to support full differentiation of neonatal rat cardiomyocytes. The alginate hydrogels have a very low porosity of well below 1 μm, while optimal porosity is more than 30 μm for angiogenesis in vivo. No differentiation of fibroblasts towards myofibroblasts has been reported in the support, even in the presence of RGD and with a stiffness of 1 kPa, which is normally optimal for differentiation towards myofibroblasts. In vivo, in the model of acute MI, the association of RGD with alginate has even been reported as being deleterious and the functional benefits on ventricular remodeling as being lost. An explanation put forward by the authors is that after functionalization of the alginate with RGD, there is a 4- to 7-fold increase in stiffness. In our study, we have not found any modification of collagen after functionalization with Sulfo-LC-SPDP and RGD [51,52,53].

#### 4.1.2. In a Solid Porous Alginate Scaffold, Functionalization with RGD Increased Contractile Differentiation, but with No Contractility

Solid microporous alginate sponge scaffolds, with a low stiffness of around 1 kPa, have been obtained by classical DE hydrothermal treatment (DHT) [110]. In these scaffolds, it has been shown that the presence of RGD is essential for the survival and maturation of neonatal rat cardiomyocytes, which also involves a stimulation of the Akt signaling. However, no contractility has been demonstrated in these experiments [110]. A combined effect is observed between RGD and heparin binding [110]. RGD increases α-SMA and inter-cardiomyocyte connexin 43. In another study, the functionalization of a solid porous alginate, obtained by DHT with RGD, improves the differentiation of neonatal rat cardiomyocytes in vitro with an increase in connexin 43. The RGD also improves the capacity of non-muscle cells to have a contractile phenotype with an increase in α-SMA expression and collagen synthesis [111].

#### 4.1.3. Possible Use of a Solid 3D Alginate Scaffold for Non-Contractile Paracrine MSC Delivery after MI

Alginate hydrogel or solid patches have been shown to improve early human MSC retention. In acute rat MI, after intramyocardial injection of free human MSCs, only 10% of MSCs are present after 24 h. Associating cells with injectable alginate or chitosan increase the retention by 14x, and by around 50x if the cells are applied as a patch of porous alginate or solid DHT collagen onto the ventricle. However, over a longer period, alginate hydrogel does not increase the cell retention rate any further, which is only 7% after 2 weeks [112].

A solid porous composite scaffold of alginate and chitosan without RGD (obtained by DHT) has been also developed, with a stiffness of 20 kPa. Applied to the infarct area immediately after MI with human MSCs, the preparation presents an improved ejection fraction (EF) by up to 1 month and a decreased local fibrosis and angiogenesis [113].

### 4.2. Best Contractility Achieved with Preparations Containing Natural Polymers Such as Collagen, Gelatin, Fibrin or Matrigel™

In 2D cultures, human cardiomyocyte cell lines (i.e., HL-1) have a better expression of inter-cardiomyocyte connexin 43 on gelatin (that has a RGD site functional) or on gelatin with fibronectin, than on collagen type I. Laminin does not induce connexin 43. In vitro, maturation of human hiPSC towards cardiomyocytes (hiPSC-CM) in a 3D environment is enhanced compared to that obtained in a 2D environment with polydimethylsiloxane (PDMS, Dow Corning) molds, fibrinogen and Matrigel™ [44].

#### 4.2.1. The Different Natural Polymers: Collagen and Gelatin

##### The Collagen Polymer: Generalities

See Figure 1 and for review [16].

Collagen can be used as a tissue graft, in hydrogels, solid gels or sponges, or in hollow micro spheres (Figure 2). It is worth pointing out that both physical and biological methods of reticulation, despite their superior cytocompatibility to chemical approaches, are very weak, often weaker than the mildest chemical approach. Furthermore, the physical methods are associated with collagen denaturation. The quest for the optimal collagen crosslinking method’s still continues. Collagen is the most abundant extracellular protein. There are many types of collagen used in preparations (e.g., mammalian/marine extract collagen, cell-produced collagens, recombinant collagens and collagen-like peptides) and in crosslinking technologies [16,92]. The prevalence of collagen in human tissues and its various inherent properties (i.e., harboring natural cell recognition signals, ability to form 3D scaffolds of various physical conformations, controllable mechanical properties and biodegradability) makes it a natural choice as a raw material for engineering most biomaterial tissues. Collagen as a natural component is very similar in different species and thus has a very low immunogenicity. For medical applications, collagen type I is mainly extracted from skin and tendons (bovine, porcine or ovine), while collagen type II is extracted from cartilaginous tissue [16,92]. Collagen in the tissue is particularly notorious for its large coherent covalently crosslinking fibrillary meshwork. To this end, different methods (diluted acidic solutions with or without enzymes, neutral salts, and alkali treatments) are used to isolate the collagen from the different tissues [16,92,114]. Collagen monomeric components interact sequentially with each other and with other ECM constituents to produce higher order structures with numerous hierarchical levels of association and function [16]. Twenty-nine different collagens have been reported so far, among which collagen I and II are the most abundant. In the heart, the collagen in direct contact with cardiomyocytes is the compliant collagen III [16]. The collagen molecule is comprised of three α chains organized in a triple helical region and two non-helical regions at the ends of the helix. Intramolecular hydrogen bonds between glycines in adjacent chains stabilize the triple helix and the hydroxyl groups on the same chain form hydrogen bonds [16]. One important feature of collagen is that it will self-assembly into more elaborate structures by spontaneous arrangement in a lateral structure or head-to-head structure to form cross-striated fibrils (500 mm in length and 500 nm in diameter) (Figure 1 panel A) [16]. Fibrillogenesis of collagen type I is dependent on temperature, pH level and ionic strength. Under appropriate conditions, collagen molecules will spontaneously assemble to form microscopic fibrils, fibril bundles and macroscopic fibers [16]. In vivo, an initial, provisional 3D fibronectin matrix has been shown to be necessary before the secondary incorporation of collagen that re-enforces the provisional fibrin matrix. Collagen spontaneously forms anarchic porous interconnected crosslinked architectures, which is not the case for gelatin. The exceptional properties of collagen (resistance to mechanical forces, temperature, degradation) are due to the triple helix. Additionally, due to the triple helix is its spontaneous fibrillogenesis. Besides its elastic and viscoelastic physical properties, mainly due to its fibers, collagen also has an exceptional resistance to shear stress. It is one of the most resistant polymers. The primary mechanical strength of collagen results from the self-assembly of collagen molecules into triple helices and collagen fibril, both of which are additionally stabilized by intra- and intermolecular crosslinks [16]. The non-collagenous components are believed to play important roles either through their unique viscoelastic properties (i.e., elastin) or via their interaction with collagen fibers (i.e., glycosaminoglycans and proteoglycans) and allow the tissue to withstand compressive and tensile forces. Compared to gelatin, collagen has a higher thermal stability. The Tm50 for tryptic measurement has been shown to be 41.5 °C for human collagen I and 39.5 °C for human collagen III. Additionally, compared to gelatin, collagen’s resistance to enzyme degradation is greater. Collagen type I is a versatile biomaterial that is widely used in medical applications due to its weak antigenicity, robust biocompatibility, and its ability to be physically and chemically modified for a wide array of applications. As such, collagen has become a major component of many tissue engineering scaffolds, drug delivery platforms, and substrates for in vitro cell culture [16]. In these applications, collagen constructs are fabricated to recapitulate a diverse set of conditions. Collagen fibrils can be aligned during or post-fabrication, cross-linked via numerous techniques, polymerized to create various fibril sizes and densities, and copolymerized into a wide array of composite scaffolds [13,78]. Approaches have been used to tune collagen to better recapitulate physiological environments for use in tissue engineering applications and studies of basic cell behavior [78].

Different techniques to control fibril alignment, methods for cross-linking collagen constructs to modulate stiffness, and composite collagen constructs to better mimic physiological extracellular matrices have been described [78].

Collagen has the ability to polymerize in vitro into a fibrillar hydrogel at physiological pH, ionic strength and temperature, following an entropy-driven process [115]. Hydrogels are water-swollen structures with properties that resemble those of soft tissues more closely than any other type of polymeric biomaterial. The intertwined fibrillar substructure is held together by electrostatic and hydrophobic bonds and entraps huge amounts of fluids, thus enabling the exchange of ions and metabolites with surrounding tissues. The flowable nature of collagen hydrogels is primarily attributed to this high liquid phase and, along with their fast assembly time (<10 min) at physiological pH and temperature, allow them to act as injectable systems and ideal carriers for cells and therapeutic/bioactive molecules [44]. Crosslinking enables control over the liquid content and influences the mechanical properties and the degradation profile of resultant hydrogels. An alternative strategy to improve the mechanical properties of hydrogels is based on confined and unconfined plastic compression [115]. These unique properties of collagen hydrogels have made them the scaffold of choice for numerous clinical indications.

Freeze-drying, also known as ice crystal templating, lyophilization, ice-segregation-induced self-assembly, or dehydrothermal treatment (DHT), is a dehydratation process that can be used for the construction of highly porous, implantable devices (Figure 3, panel I). DHT is a very interesting approach because it allows the fabrication of a highly porous scaffold at the same time as provoking, during the heating phase of the procedure, a mild physical crosslinking of the scaffold. During the first step of DHT treatment, the preparation is maintained at below 0 °C and the collagen is entrapped within the developing ice crystals, which form hexagonal structures. By adjusting the level of freezing below 0 °C and the speed of freezing, it is possible to control porosity [93,116,117]. More specifically, primary freezing at higher temperatures increases the pore size through the formation of large ice crystals, whereas freezing at lower temperatures decreases the pore size through the formation of small ice crystals. It is also possible to create a temperature gradient during this phase to obtain a DHT scaffold with a particular orientation [93]. The second step of the DHT treatment is the sublimation step, induced by the immediate application of a temperature above 100 °C in a low-pressure environment that will provoke the sublimation of the ice crystals. Dehydration provoked by DHT above 100 °C provokes condensation reactions that induce the formation of interchain crosslinks as a result of condensation reactions either by amide formation or esterification between carboxyl and free amino and hydroxyl groups, respectively. Condensation is facilitated by removal of water molecules in a vacuum oven (low pressure) [16]. The effect of temperature (120–160 °C) and the duration of heating have also been investigated and are found to impact on scaffold stiffness and biocompatibility [118]. For optimal bioactivity, the pores should be large enough to permit the migration of cells and the diffusion of nutrients, small enough to promote cell attachment [119]. However, pores should not be too small, as they could restrict cell attachment and differentiation potential [120,121]. Condensation is facilitated by removal of the water molecules in a vacuum oven. DHT does not appear to reduce the availability of cell-adhesive ligands within collagen [122].

As with other polymers, different techniques have been developed to control collagen stiffness. These techniques can be broadly classified into chemical methods (such as crosslinking with glutaraldehyde, EDC/NHS, genipin), physical methods (such as physical reticulation during dehydrothermal treatment (DHT [93,116,117,118,123,124]) and enzymatic methods (such as treatment with transglutaminase [16]). The glutaraldehyde is toxic for the cell. EDC (carbodiimide) is less toxic, but disturbs adhesion sites on collagen [125]. The same is true regarding the toxicity of genipin for high concentrations of more than 10 mM. Chemical reticulations in general are associated with increased stiffness, lower viscoelasticity and decreased adhesion site availability. Another physical method (UV) has been shown to be cytotoxic, to also cause partial fragmentation of collagen, and to require transparent scaffold for homogenous crosslinking [16].

Several methods have been developed to improve collagen polymer parameters. In native tissue, the collagen is associated with proteoglycanes. In some applications, the collagen has been associated with glycoamino glycans or polysaccharides such as chitosan [78]. No contractility has been demonstrated so far in such scaffolds. Several classical methods to fabricate solid scaffolds with polymers have also, so far, been disappointing. Although the benefits of electrospinning are now well known, unfortunately the electrospinning of collagen still remains a challenge, as the current process leads to irreversible denaturation and scaffolds obtained by electrospinning are very stiff (around 100 kPa) and have a very low porosity. Moreover, no true contractility has yet been demonstrated in these scaffolds [78,94]. An emerging method to fabricate collagen scaffolds of sufficient stiffness uses mechanical compression [16], but the porosity of these scaffolds is very low compared to that obtained with the previous method. Enzymatic crosslinking (i.e., transglutaminase) is generally of very poor quality [16]. Compressed collagen patches have been used with growth factors applied onto the epicardium in the context of acute MI [126].

##### The Gelatin Polymer: Generalities

See Figure 1, panel B and for review [15,16].

**Figure 4:**
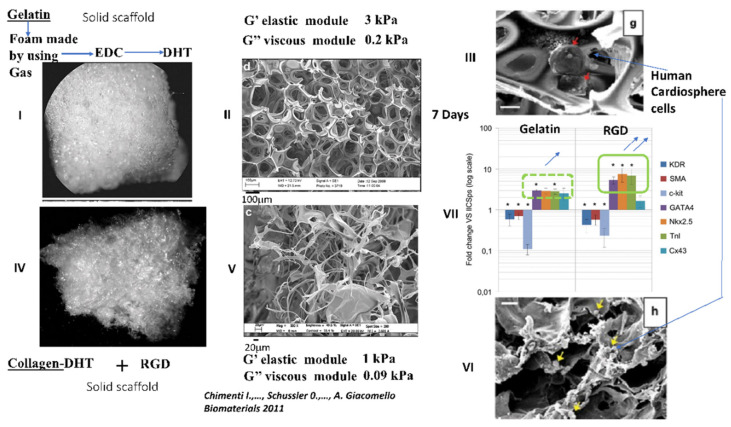
Comparison of the behavior and differentiation potential of “human Cardiospheres” towards cardiomyocytes in vitro, after seeding in solid DHT gelatin (panels **I**, **II**, **III**) or solid collagen DHT functionalized with the RGD peptide (panels **IV**, **V**, **VI**). The solid gelatin scaffold has been obtained by firstly fabricating a porous foam by insufflating gas in gelatin solution and then through chemical reticulation by EDC to maintain the architecture (as one of us has previously reported [127]). To increase the porosity of the preparation, the foam underwent further DHT procedures, as reported earlier [127] (Panel **I**). The preparation has a low stiffness value of around 3 kPa (Panel **II**). The solid scaffold (Panels **IV**, **V**, **VI**) has been prepared using a commercial collagen scaffold obtained by DHT (i.e., Ultrafoam™ from Bard) and functionalized with the RGD peptide, as we have previously reported [48]. The stiffness of the collagen after functionalization with the RGD is around 1 kPa (Panel **V**) [128]. As previously reported, and as shown in panel **III**, “human Cardiospheres” in a solid gelatin scaffold stay as a cluster, while in solid collagen RGD, the structure “as cluster” is lost and the cells migrate through all the scaffold. Cardiomyocyte markers, GATA4, Nkx2.5 and troponin, are 3 times higher in collagen RGD than in gelatin-based scaffolds. Thus, for engineering a contractile tissue, the collagen RGD preparation seems to be better than that with gelatin [128].

Gelatin is a molecular derivative of collagen obtained through the irreversible denaturation of collagen proteins by means of high temperatures [15]. Gelatin isolation involves “harsher” methods, employing heat associated with acid or alkaline treatment to isolate and denature collagen to gelatin A (acid-based) or B (alkaline-based), both of which contain single broken-down triple helices [15]. High temperatures of more than 40 °C during isolation provoke a partial denaturation and reversible disruption of the quaternary structure of collagen. Digestion by pepsin enzyme has been shown to enhance recovery, but it definitively alters the triple helices [15]. Enzymatic digestion additionally gives rise to irreversible small linear peptides and the quaternary structure is definitively lost [15]. Gelatin shares a very close molecular structure and function with collagen. During the transformation of collagen to gelatin, RGD adhesion sites become functional but, at the same time, the main binding site on collagen GFOGER (Gly-Ph-OHPro-Glu-Arg) for integrins is lost. Gelatin is generally produced by irreversible hydrolyzation of the triple helical structure of collagen through processes such as heat and enzymatic denaturation, producing random coiled domains. Gelatin has less organization and fewer structural properties than collagen, but has a very similar molecular composition. The composition of gelatin has been shown to be 21% Glycine, 12% Hydroxyproline, 12% Proline and other amino acids such as Alanine, Arginine, Aspartic acid, Lysine, Leucine and Valine. The RGD is present on gelatin and functional because of the loss of the helices that are making the RGD non-functional on native non-denatured collagen [15]. One important intrinsic property of gelatin is that in solution, in sufficiently high concentrations, it forms a semisolid gel at low temperature. Gelatin solution is thought to become a random coil conformation when the temperature goes above 40–50 °C. As the gelatin solution cools to below 30 °C, a reverse coil to triple helix transition occurs and natural hydrogen bonds stabilize the conformation [15]. However, the process is reversible and needs stabilization by a further reticulation. Besides concentration and temperature, another factor influencing the gel strength is the pH value, especially between 3.0 and 6.0. Changes in pH cause ionic changes between gelatin monomers and consequently affect the hydrogen-bound spontaneous crosslink process. It has been shown that gelatin forms helices more easily at around pH 5.0. The high spontaneous propensity of collagen monomers for complex polymerization is lost with gelatin preparations, which consequently have a lower porosity. DHT or preparations of gelatin such as foam by creating artificial pores through mixing with microparticles such as sugar, paraffin or gas injected directly into hydrogels, may help to partially recover porosity [127]. The gel of gelatin needs to be stabilized by physical or chemical reticulation. DHT treatment of gelatin gels is a way of stabilizing the preparation and increasing its porosity [127]. We have implanted human cardiospheres in DHT gelatin foam or in DHT collagen with RGD in vitro (Figure 4) [128].

Although the stiffness of each of the preparations is almost the same [128], human cardiosphere cells in gelatin-based preparations do not migrate and their cardiogenic differentiation is lower than in the solid collagen scaffold obtained by DHT and functionalized with RGD (Figure 4) [128].

#### 4.2.2. Contractility in Gelatin Hydrogel Associated with Fibrin or PMDS and Neonatal Rat Cardiomyocytes

In vitro, gelatin has recently been used as a 3D hydrogel to engineer a true contractile tissue using 3D bioprinting technology with neonatal rat cardiomyocytes associated with fibrin and hyaluronic acid [67] or with poly(ethylene-glycerol-polydimethylsiloxane) (PMDS) [68]. A research has demonstrated the possibility, in vitro, of using the promising 3D printing technology to engineer a hydrogel with neonatal rat cardiomyocytes with gelatin. Preparations containing gelatin, in association with fibrin and hyaluronic acid, developed a force of 2 mN, and still with a low scaffold thickness (i.e., 0.6 mm) [67]. Human cardiac-derived progenitor cells have been associated with a 3D printed solid patch of gelatin/hyaluronic acid, and crosslinked with a thiol linker and applied immediately onto the infarct area in a mouse model. Cells survive for up to one month with some partial differentiation of the contractile apparatus. Preparation improve early heart remodeling, but there is no effective mechanical connection between the patch and the native myocardium [129].

#### 4.2.3. Contractility in 3D Gels of Collagen, Fibrin or Matrigel™ with Neonatal Rat Cardiomyocytes or Human Cells

See Figure 2.

Up to now, the best contractility has been obtained with thin strips of neonatal rat cardiomyocytes in collagen type I gels and cells embedded in a gel of tumor basement membrane (i.e., Matrigel™), using chronic physical stimulus (mechanical or electrical stimulation) and high horse serum (Figure 2, panel VI). However, the use of Matrigel™ presents serious limitations since, as a gel, it fills the pores of the scaffolds and modifies cell interactions with the collagen scaffold. Matrigel also disturbs nutriment diffusion, so that the use of a bioreactor is mandatory, which directly reduces the maximum thickness of the construct. In vivo, Matrigel™ is very immunogenic and the animal needs to be under immunosuppression. More recently it has been shown that the use of Matrigel™ could be replaced by thyroid hormone T3 at the beginning of the protocol [130]. The authors report the development of a force of around 78 mN/nm^2^ [131] or 2 mN [132]. The exact role of T3 needs to be evaluated, but an RGD binding site on the T3 thyroglobulin interacts with αvβ3 and αvβ5 in tumor cells. Thyroid hormones have been shown to increase contractility. T3 promotes the maturation of hiPSC-derived cardiomyocytes.

More recently, in fibrin or collagen gels, contractile tissues have been developed with human cardiomyocytes derived from embryonic cells (ES or hiPSC) (Figure 2, panel V) (for reviews [38]). Contractility has been demonstrated in collagen type I gels or fibrin gels seeded with human contractile cells. Protocols for the differentiation of cardiomyocytes or myofibroblasts derived from human embryonic stem cells have been documented. In a study, the derived cells have been incorporated into collagen type I gel and then undergo mechanical and electrical stimulation. Unlike for neonatal rat protocols, the use of Matrigel™, T3 or insulin is not necessary. The addition of laminin or fibrin does not improve contractility. Optimal contractility has been observed for a preparation with 70% of cardiomyocytes and 30% of fibroblasts. It is possible to engineer a large construct of 35 × 34 mm with a thickness up to 0.5 mm. These constructs contain 40 × 10^6^ cardiomyocytes and develop forces of 1.5 mN under optimal conditions at 6 weeks [79]. In another study, collagen type I gel with human-induced cardiomyocytes has been transferred onto a stretcher for functional mechanical maturation during additional 12–14 days. If T3 and insulin are used for at least 24 h after the casting, the use of Matrigel™ is not necessary. The stretching allows terminal cardiomyocyte maturation of the contractile apparatus with an average force of 0.35 mN “by loop”. The construct is able to be transported for 2–3 days at 21 °C and then implanted in a rat model of chronic infarct heart (1-month old). In the first few days, there is a rapid alteration of cellular viability with less than 20% of cells that survive in the long term. The preparation improves ventricular remodeling, but not contractility. The effect is independent of cell survival, suggesting again a paracrine effect [108].

Contractility has also been demonstrated in 3D fibrin gels seeded with human contractile cells. Patches with human hESC have been developed in fibrin gel in vitro with a better contractility for again mixed preparations of cells derived from hESC with 70–80% of cardiomyocytes (hESC-CM). These preparations with fibrin gels survive and become electrically incorporated after transplantation onto a large swine infarct. The presence of a cellularized patch decreases the infarct size and increases neoangiogenesis in the peri-infarct region [30]. Moreover, the fibrin gel is associated with a synthetic polydimethylsiloxane (PDMS) polymer to control the 3D microarchitecture. Maturation of hiPSC-CM is obtained if the structures are electrically stimulated, but no true contractility has been demonstrated [107]. In vivo, the survival and electrical incorporation of a thin patch of human hiPSC-CM in a construct of PDM, fibrinogen and Matrigel™ and epicardially applicate has been demonstrated in nude mice [106].

#### 4.2.4. Interesting Long-Term Contractility and Thick Tissue Obtained In Vitro in Solid Porous DHT Collagen Scaffolds

The most advanced 3D constructs in terms of tissue structure and long-term functioning have been obtained with neonatal rat cardiomyocytes seeded in solid collagen sponges, with chronic electrical pacing and Matrigel™ with the mandatory use of bioreactors (Figure 2, panel VI) [41,43]. A classical easy way to obtain a solid collagen scaffold is to use a physical de-hydrothermal type of production that also allows a mild physical reticulation of the scaffold at the same time. Highly porous collagen sponges with a pore size between 20–200 micrometers allowing free nutriment diffusion and favoring cell colonization can be achieved [128]. During the process of DHT, it is also possible to create a temperature gradient to obtain an oriented collagen scaffold that promotes the maturation of contractile cells (Figure 3, panel B). The electrical mapping of the solid DHT collagen has been investigated and homogenous conductibility has been reported [133]. Radisic et al. have demonstrated how a 3D collagen non-oriented porous scaffold, used as a clinical hemostatic (Ultrafoam™, Bard), can be employed for the engineering of a contractile tissue when seeded with neonatal rat cardiomyocytes [41]. However, for cell seeding and survival, a gel obtained from tumors (i.e., Matrigel™) is necessary. In addition, the use of Matrigel™ inside the solid DHT scaffold limits nutriment diffusion and a perfusion bioreactor is therefore necessary. For cellular maturation, the use of chronic electrical stimulations is also necessary. In the Radisic et al. experiments with neonatal rat cardiomyocytes seeded in DHT scaffold with Matrigel™, the stimulation threshold is around 3 V/cm [41]. We have performed the same experiment with the same solid DHT collagen scaffold but functionalized with the RGD, and without the use of Matrigel™, bioreactors or chronic electrostimulation, and we have been able to engineer a very efficient contractile patch with a lower threshold of around 1 V/cm [48]. This last stimulation threshold is within the range of the natural stimulation threshold in the heart. With the mouse myoblast cell line C12C12, it has been shown that electrical stimulation induces a contractile apparatus maturation in this solid collagen DHT [134]. Collagen functionality can be probably improved by chemical functionalization with the key RGD peptide that is not functional on natural collagen [48]. In the scaffold functionalized with RGD, and without Matrigel™, perfusion and electrical stimulation, we have reported a contractility of 90 μN with RGD, and around 30 μN without it [48]. In addition, the stimulation threshold for the RGD collagen preparation was very low, below 1 V/cm for RGD, and around 3 V/cm without it [48]. We have been able to engineer a contractile patch with a thickness of a 3 mm. Earlier we have reported the very high porosity and low compliance of a collagen patch of around 1 kPa [51,128] that is not modified by functionalization with the RGD peptide, thanks to the peptide functionalization method that we have developed [51,52,53].

For optimal cardiac tissue, it is important that the scaffold promotes the survival and differentiation of other cardiac cell types, such as endothelial cells and MSCs. Our research [51], and those of others [54], have reported that MSCs seeded in solid DHT collagen present increased paracrine and regenerative properties. We have also demonstrated, in vitro, how human multipotent stem cells (MSC) seeded in a RGD collagen scaffold in the presence of a classical culture medium for expansion (rich in plated lysate containing TGFβ) massively differentiate towards contractile myofibroblasts [51,52,53]. The functionality in terms of paracrine activity is stronger than in 2D cultures and is not altered during differentiation [51]. We have also reported how solid collagen RGD scaffolds promote the differentiation of human cardiac cells (e.g., “Human Cardiospheres”) towards cardiomyocytes [128]. Moreover, the RGD scaffolds, compared with collagen, enhance the differentiation of mouse embryonic stem cells (EBS 5 days) towards cardiomyocytes if seeded in the same collagen RGD scaffold [135]. The development of angiogenesis is essential for tissue engineering and survival following implantation, as well as for optimal contractility in vitro. A functionalization of the DHT scaffold with VEGF and/or angiopoietin 1 has been proposed by Radisic et al. [136,137]. Again, growth factors need to be covalently bound to the collagen scaffold for efficiency. The RGD is crucial for interaction with endothelial progenitor cells at vasculature sprouts during angiogenesis. The solid collagen DHT scaffold has been used to replace the entire thickness of rat right ventricle for up to more than one month [136]. The functionalization of collagen patches with VEGF induces higher neoangiogenesis and maintains patch thickness [136].

Our group has been the first to demonstrate in mice [45] and in humans [46,47] that a solid porous collagen scaffold, cellularized with human bone marrow and applied to recent chronic infarcts in association with an intramyocardial injection, obtains more satisfactory results than free intramyocardial cell injections alone.

### 4.3. Contractility with Decellularized Tissue Containing Collagen

For review [138,139].

#### 4.3.1. Classical Methods for Obtaining a Decellularized Matrix Tissue and the Limits of This Matrix

The decellularizing process is obtained by chemical (SDS detergent-based method), enzymatic (Triton X-100) or physical treatment of the tissue to remove the cells while preserving most ECM components and organization as far as is possible (Figure 2, panel II) [139]. It has been shown that each organ-specific ECM promotes maturation of specific progenitors and that the resulting natural cardiac ECM is the most promising matrix for cardiac cells [139]. Important limitations of decellularized ECM are their low porosity, limiting cellular colonization and nutriment diffusion, and their ability to preserve native ECM component stoichiometry, such as basal lamina versus structural protein fractions. Reproducibility between different donor hearts remains a significant challenge, as does the higher immunogenicity of these matrices compared to that of reconstituted collagen [139].

In an effort to improve the mechanical properties of decellularized ECM-based products (Figure 2, panel II), a composite scaffold of soluble decellularized ECM with chitosan and alginate has been recently examined and found to enhance human MSC proliferation, but no contractility has been demonstrated. Crosslinking of decellularized ECM with a chemical reagent (i.e., genipin) was shown to impair cell matrix interaction with decreased α5 and β1 in associated MSCs. A tendency for bone differentiation is also observed.

#### 4.3.2. True Contractility with Shortening and Developed Forces Have Been Demonstrated Only on Thin Sections of Decellularized Tissue

See Figure 2, panel II.

Three-dimensional decellularized extracellular matrix preparations have been shown to be superior to 2D preparations for cardiac differentiation [140]. In 3D constructs, differentiation of iPSC-derived cardiomyocytes is enhanced with an increased beating activity. However, no true contractility has been demonstrated [140]. A thin section (150 μm) of decellularized heart matrix is seeded with human-derived cardiomyocytes from hESC-CM or hiPSC-CM for 3 weeks. Homogenous cellular colonization of the full scaffold thickness is observed. Peak twitch stress is measured at 0.49 mN/mm^2^, but with a low thickness value. While some progress with human ECM-based scaffolds has been made [141], the contractility of the resulting fECTs is very low, with generated forces in decellularized scaffolds and whole heart amounting to only 100 μN [141].

## 5. Possible Engineering of a Large Contractile Patch with Human Cells (hESC-CM) in Gels of Matrigel™, Fibrin or Collagen

For review [28].

Recently, a patch with hESC-CM has been implanted in a chronic infarct heart in macaque. The scaffold used is a Matrigel™ gel. The preparations have been mechanically stimulated for 12–14 days before implantation. In immunosuppressed macaque, the preparations survived for more than 220 days with a well-organized mature sarcomere alignment. A limitation is the presence of ventricular arrhythmias in 100% of the animals. In vivo, further maturation of hESC-CM is reported along with electrical coupling between native macaque cardiomyocytes and human ESC-CMs. It is not possible to use a Matrigel™ preparation in humans [109]. Optimizing the stimulation threshold of the preparation may decrease the prevalence of arrhythmia.

In another study [30], a fibrin gel is used with different types of human cells derived from ESC (embryonic stem cells) and result in the development of ESC-CM (cardiomyocytes), endothelial cells and smooth muscle cells. Preparations are mechanically stimulated in vitro. Patches of up to 1.25 mm in thickness are developed for a period of 7 days before implantation. Stability of the preparations for a longer period is not evaluated, but gels are known to present very poor mechanical properties. The patches were implanted onto the MI zone very early after MI (60 min.). No arrhythmia is reported with this composite patch. However, only 10% of the cells survived in the patch. Nevertheless, the patch still improves left ventricle contractility, mostly by a paracrine activity in the border zone [30]. Cardiopatches of hiES-CM have been developed in collagen gel type I with mechanical stimulation for 12–14 days. The preparation can be shipped at ambient temperature and applied to the left ventricle in models of chronic heart ischemia. Overall, 25% of the cells in the patch survives for a long period of 220 days [108].

## 6. Rationale for Using Collagen or Gelatin as a Polymer Backbone for Tissue Engineering 3D Scaffolds and the Limits of Hydrogels and Gels

### 6.1. Rationale for Using Collagen Type I or III as a Polymer Backbone

Collagen is the major constituent and structural component of the ECM, including the cardiac extracellular matrix, and is therefore an obvious choice as a scaffold material for regenerative medicine. Collagen monomers have a natural propensity for spontaneous fibrillogenesis and for forming porous 3D structures. Collagen provides a biomimetic environment for cell growth, since it has a fibrous structure, appropriate mechanical properties (stiffness/viscoelasticity and resistance to deformation) and some important adhesion molecules. In addition, it is highly biocompatible and biodegradable.

The cardiac ECM is composed of structural collagen type I (80%), collagen type III (10%) and non-structural proteins (10%), such as collagen type IV, laminin, or fibronectin. Collagen type I fibrils provide the majority of the tensile strength, while the addition of collagen III to collagen I is found to increase tissue elasticity [100,142,143,144]. Collagen type III is very flexible compared to collagen type I [145] and is the main collagen component surrounding the cardiomyocytes. Fibronectin, containing a RGD motif that is critical in its functionality, is ubiquitously present in the ECM of normal myocardium and surrounds the cardiomyocytes [145]. Laminin has been shown to be secreted on collagen by cardiomyocytes and to directly control the initial sarcomere Z band organization and development [17]. The fibronectin has the same distribution as laminin in the ECM and probably reinforces the maturation of the contractile apparatus.

### 6.2. Limits of Gelatin as a Natural Polymer Backbone

Gelatin is a thermally denaturized collagen and possesses a more disorganized structure than normal collagen, which considerably alters the material mechanical stability. Denaturation alters the macromolecular order in collagen, but the chemical composition is largely maintained. With gelatin, the tendency of natural collagen towards spontaneous fibrillogensis is lost, and engineered scaffolds have a reduced porosity. At the same time, in random gelatin, the important binding site “RGD” is available, while it is not functional in native collagen [146].

The possible use of different amounts of several components such as collagen, gelatin and elastin to design soft material has been investigated in the presence of fibrosarcoma cell lines. To maintain large pore size for cellular colonization, chemical crosslinking by carbodiimide is necessary to maintain structural stability, strength and resistance to degradation in all the scaffold preparations mentioned above [147]. In vivo, results with gelatin-based scaffolds are very poor when compared to collagen-based scaffolds. There is an early, very extensive infiltration of the biomaterial after implantation in the muscle. The quantity of angiogenesis in the gelatin is increased for up to one month. However, then there is a fibrotic scar with very poor angiogenesis [146]. That is not the case with biomaterials made with native collagen [146].

### 6.3. Structural Limits of Collagen or Gelatin Hydrogels and Gels for Contractile Tissue Engineering and the Necessity for Reinforcement by Reticulation or Association with Structural Polymers

#### 6.3.1. Limits of Contractility Reported in Hydrogels of Gelatin or Collagen

Collagen type I scaffolds have several limitations when used as hydrogel for cardiac tissue engineering: (1) they are not easily remodeled to yield physiological cell density and connectivity; (2) they do not stimulate endogenous matrix secretion by cardiac cells; and (3) they are stiffer than other hydrogel scaffolds. As a result, collagen I does not provide ideal conditions for cardiomyocyte maturation and macroscopic contractions. Furthermore, collagen I hydrogels have a long gelling time which can lead to a settling of seeded cells during polymerization, yielding a non-uniform cell density.

Gelatin hydrogels have poorer structural properties than collagen hydrogel and thus need to be reinforced. Specifically, gelatin methacrylol (GelMA) hydrogels have tunable biophysical and biochemical properties and have been increasingly used for the generation of contractile cardiac tissue. However, the functional properties of cardiomyocytes embedded in GelMA hydrogels remain inferior to those in collagen type I and fibrin-based tissues. In hydrogel of gelatin-PEG, the 3D structure has revealed an over-expression of β1 integrins on human cardiac stem cells and optimal differentiation for 8 kPa, not 2 kPa.

#### 6.3.2. Limits of Contractility Reported in Gels of Gelatin or Collagen

The main drawbacks of collagen gel are its limited stiffness, poor mechanical quality and a low porosity that is not optimal for nutriment diffusion. In addition, the mechanical properties of the gel rapidly degrade in vitro. If not functionalized with the RGD peptide, collagen type I may therefore not be an ideal polymer, due to the lack of functional RGD sites. When coated on collagen type I in 2D cultures, the collagen failed to induce IES differentiation towards cardiomyocytes. In 3D collagen gel type I, different levels of stiffness have been tried [148], but the contractility of neonatal rat cardiomyocytes is impaired for all the stiffness levels [149].

In 2D and 3D constructs, human myoblasts have been shown to interact with the RGD motif (αvβ3 and αl5β1) in gelatin, but not in collagen [100]. Denaturation of collagen to gelatin is accompanied by the loss of the collagen main adhesion sequence (i.e., GxOGER) ligand for integrins and the appearance of the cryptic RGD. It has been suggested that associating a certain proportion of gelatin and collagen can improve the biofunctionality of the preparation. In mixtures of gel of collagen with gelatin, a decreased availability of GxOGER has been observed, along with an increased availability of RGD [100]. Not surprisingly, the association has a significant effect on the scaffolds’ mechanical properties, which become considerably inferior to scaffolds made with collagen only and, more unpredictable [100].

Several techniques have been developed to improve gel physical properties, chemical reticulation, and the association with other synthetic or natural polymer components. The limits of collagen’s chemical reticulation have been shown with alterations of its biological functionality by a significant disturbance of the recognition motif for integrins and further limitations of gel porosity. In addition, the fibrillar network that improves interaction with associated cells is not spontaneously present in collagen gel [58]. Compared with a scaffold of just collagen alone, the addition of gelatin reduces both scaffold stiffness and degradation time. The reticulation of the entire scaffold is essential for structural stability, strength, and resistance to degradation [147]. The chemical reticulation of the scaffold is performed with a chemical reticulating agent EDC was evaluated. The stiffness of the scaffold made of just collagen alone is too high (around 80 kPa) as opposed to around 4.6 kPa for gelatin alone [147]. Chemical reticulation of gelatin and collagen with EDC has been shown, in 2D and 3D, to alter the RGD and GxOGER sites in gelatin and collagen [100]. It has also been shown that after chemical reticulation of collagen type I with EDC, it is possible to partially recover the functionality by further functionalization of the collagen with the oligopeptides (i.e., GFOGER) [101].

Long-term maintenance of myofibroblasts in collagen gel has been shown to require the gel being attached and thus being stretched. In our in vitro study of a solid collagen scaffold with low stiffness, we have been able to induce and maintain myofibroblasts from human MSCs for several weeks, without the need for stretching the scaffolds [51]. Recently, collagen type I gels not functionalized with RGD but in association with Matrigel™ have been shown capable of sustaining human hESC-CM maturation in vitro. The presence of MSCs, fibroblasts and mechanical stress improves the contractile maturation of the preparation. In this experiment, stress increases in the integrins for the RGD motif β1, αv and α5 [150].

## 7. Rationale for Having a Solid 3D Scaffold Obtained by Physical Reticulation Instead of Chemical Reticulation

In the tissue, the cell regulates the ECM by intrinsic natural crosslinking (transglutaminase) or by recruitment structural multivalent proteins that reinforce the tissue. In vitro, however, physical properties of reconstituted collagen can be improved by crosslinking: physical, chemical or enzymatic. Reticulation is a way for improving material stiffness, in hydrogels or gel and to limit material degradation and to maintain material 3D structural properties, such as porosity and orientation, and ultrastructural properties. However, reticulation also decreases scaffold viscoelasticity.

A common form of reticulation is chemical reticulation, in which the agent used should not be cytotoxic initially or at the time of degradation. However, most chemical reagents are cytotoxic, although some have a lower toxicity, such as EDC/NHS or genipin. Collagen product crosslinking by chemical reagents most often occurs on a free –NH2 amine group present on the collagen. These sites are limited and are also used for collagen functionalization with adhesion molecules. A crosslinking step is often used to stabilize the mechanical and degradation properties of materials, but has a major drawback in its detrimental effect on cell biology. Chemical crosslinking of collagen film with EDC/NHS to modify substrate stiffness is accompanied by the loss of reactivity towards the important collagen-binding GFOGER motif for α1β1 and α2β1 [125]. Functionalization of collagen films with the main adhesion molecule (GFOGER) partially restores the collagen functionality after EDC/NHS reticulation [101,151].

During the fabrication of 3D collagen scaffolds, such as the DHT scaffold (Dehydrothermal treatment), a mild physical reticulation occurs at high temperatures. For the purpose of tissue engineering, the use of mild physical reticulation is superior to chemical reticulation because it preserves most of the biological properties of the polymer. In addition, many chemical reticulating agents are toxic for the cells. We have also reported the possibility of using a physical rather than chemical reticulation of collagen to obtain a scaffold that is solid and yet maintains a low stiffness value (around 1 kPa) [51,52,53]. In this scaffold, we [48] and other researchers [41] have demonstrated the possibility of forming a very efficient contractile construct in the presence of neonatal rat cardiomyocytes. We have also compared, in vitro, the differentiation capability of “human cardiospheres” seeded in 3D gelatin scaffold (i.e., “foam”) crosslinks with EDC to improve mechanical properties and in 3D collagen type I/III scaffolds, obtained by DHT and thus with a mild physical reticulation (Figure 4). While the stiffness of both scaffolds is low and of a similar value (i.e., around 1 kPa), the differentiation of human cardiospheres towards cardiomyocytes is 3 times higher in the RGD scaffold than in the gelatin one, and the expression of Cx43 intercardiomyocyte junctional proteins is enhanced [128]. After seeding human “cardiosphere clusters” in the different collagen RGD and gelatin solid scaffolds, the cluster stays intact in the gelatin-based scaffold while in the collagen-RGD scaffold the cluster organization disappears and the cells migrate, resulting in an equal distribution of cells throughout the scaffold [128]. Collagen-RGD can be therefore more appropriate for inducing cardiosphere cardiac differentiation and for engineering a contractile tissue [128]. Again, the additional chemical reticulation of the solid DHT scaffold made of collagen and chondroitin by chemical reagent EDC is associated with important changes in the 3D architecture [152].

## 8. Functionalization of Biomaterials with Peptides Such as RGD

For review [65,153,154].

The different ways used in cardiac tissue engineering to achieve functionalization of synthetic materials have been reviewed [154,155]. Numerous materials have been functionalized with the RGD [61]. There are two strategies for the biofunctionalization of polymers. The first is the pre-polymerization functionalization via polymerization of functional monomers (e.g., alcohols, carboxylic acids, amines, and acrylates). This procedure provides, for example, functional polyesters or polyurethanes with a defined chemical structure that allows further modifications following polymerization. The second strategy is a post-polymerization functionalization, which is the modification of the polymer after the polymerization process. Post-polymerization techniques might be specific, targeting functional groups present in the polymer via carbodiimide or UV-initiated radical coupling, or non-specific, using azide- or glutaraldehyde-based coupling. A disadvantage of the non-specific covalent functionalization method is that it may result in the destruction of biomolecule bioactivity and/or can involve side reactions such as hydrolysis, chain-degradation, or cross-linking. Different techniques for the addition of RGD peptide during scaffold fabrication, such as electrospinning or 3D printing, have been documented [153].

While the functionalization of synthetic polymers that lack all adhesion molecules is logical, it appears that, in natural polymers, the associated cells often secrete their own membrane basal ECM protein, so that functionalization is not always necessary. Neonatal rat cardiomyocytes on natural collagen type I have been shown to synthetize their own basement membrane of laminin [17] and, in a more recent study, adult rat cardiomyocytes in vitro have been shown to synthetize collagen type VI and laminin [17]. In many cases, the functionalization of scaffolds with oligopeptides corresponding to the binding site of integrin receptors can recapitulate the full protein activity [67,76,79,156]. The main proteins of the ECM are collagen, vitronectin, fibronectin, laminin, and collagen type I. All these proteins, in terms of biological signaling, can be replaced by short peptides (namely RGD for vitronectin and fibronectin), IKVAV and YIGSR for laminin, and GFOGER for collagen [61].

The mere absorption of the ligands on the surface leads to unpredictable, nonspecific and potentially unstable interactions with both the cell and the material surface [61]. For correct integrin activation and clustering, the cell needs to provoke a traction force on the ligand. If the ligand is not fixed to the ECM, the correct activation does not occur [12,61,65]. High forces are exerted on the interface and this enhances cell interactions with the formation of integrin receptor clustering and focal adhesion complexes (FAK) [12]. The absence of covalent fixation and possible peptide internalization induce a deleterious effect on the cells [61]. Much better results are obtained with chemical linkers. The interactions between cells and materials is based on several parameters, among which the densities of integrin binding ligands, anchorage and presentation on the surface are indispensable factors [12]. For optimal efficiency, RGD should be bound and presented by a spacer arm of around 30-40 Angstroms [61]. For optimal interaction with human MSCs in poly(ethylene glycol) (PEG) hydrogel, it has been shown that the RGD should be covalently bound. However, the soluble RGD has the opposite effect. The RGD bonded to this last hydrogel increases the expression of αvβ3 in associated MSCs to 90% positive, while only 10% are positive in the absence of RGD. Maximum interpeptide distance has been shown to be 50 nm. However, this can increase up to 200 nm depending on substrate rigidity. In 2D environments, the minimum distance only influences the bioactivity in regular patterns. Optimal density in 2D is 50 pmol/cm^2^. A branched peptide enhances the bioactivity by promoting integrin clustering [12].

## 9. Functionalization of Solid Collagen Scaffolds with the RGD Peptide

Modifying strategies to target a uniquely reactive amino acid can be a particularly powerful step. There are several strategies for the coupling or conjugation of biomaterials with oligopeptides, including chemical modification, enzyme mediated conjugation, photo conjugation and activation, photocaging and activation of reactive functionality [63].

Methods used for the functionalization of scaffolds with the RGD peptide have been reviewed [61]. Glycosaminoglycan collagen matrices have been shown to be enhanced by RGD derivatization [107,152,157]. For collagen, functionalization mostly occurs on the –NH2 sites present on lysine, glutamate and aspartate amino acids. The number of amine sites is limited. We have developed a very safe method for collagen functionalization with GRGDS peptide in 3D porous solid scaffolds by means of heterogeneous solid phase synthesis and also demonstrated an optimal presentation by introducing a 36-angstrom spacer. The stiffness of the scaffold is not modified by functionalization with the RGD peptide [51,52]. Although the substitution of biomaterial is supposed to be simple, the functionalization of alginate with the RGD peptide, which is a classical type of scaffold for cardiac tissue engineering, results in a total loss of its bioactivity due to a 4- to 6-fold increase in stiffness [158].

The peptide is unidirectionally substituted on a free –NH2 site present on collagen. The water-soluble Sulfo-LC-SPDP is used to modify the –NH2 site on the collagen and on the –NH2 site on the glycine of the GRGDS peptide. The derived collagen is reduced, separately washed, and then reacts with the other reagent [48]. An amide bond is formed. During the final step of the modification process, a thiol group is liberated in the medium, a process that can be measured for monitoring and quantifying of the covalent fixation. We have also demonstrated the introduction of a flexible spacer arm of 36 angstrom, which has been shown to be optimal for RGD peptide presentation [38].

More recently, we have demonstrated how this scaffold, functionalized with the RGD, can be used for the differentiation of mouse embryonic cells towards cardiomyocytes [135], the differentiation of “Human Cardiospheres” towards cardiomyocytes [128] and the differentiation of human MSCs towards contractile myofibroblasts [51,52,53]. We have also shown, in vitro, how functionalization with the RGD peptide enhanced contractility by improving actin/myosin crossbridges in human myofibroblasts derived from hMSCs [53]. Due to their paracrine functionality, MSCs are important cells for delivery after MI. We have also found that human MSCs in this solid collagen DHT scaffold, with or without RGD, preserve their paracrine function and their immunosuppressive capability and that this is even enhanced compared with classical two-day cultures [51]. Human MSCs in the collagen DHT have also been recently assessed by the research group of Radisic M. et al. [54].

## 10. Positive Effects of RGD on Most Biomaterials for Limiting MI Size and for Heart Cell Therapy

Limited success in cell therapy is possibly due to poor initial cellular retention and survival in hypoxic and inflammatory environments. The application of cells through a patch in contact with the epicardial layer, and not in the infarct zone, may be a way of optimizing the treatment of the epicardial layer and border zone where generation takes place. It has been shown in large animal models of ischemia that epicardial cell application is better than intramyocardial injection in treating the epicardial layer [159]. The association of the cells with 3D scaffolds functionalized with the RGD can be a way for improving cell retention and survival [29].

Due to their paracrine functionality and safety, MSCs are one of the most promising cell groups for cell therapy in the heart. The transplantation of MSCs in collagen patches or in decellularized tissue applied onto the contractile muscle has failed to prevent cell migration. In peripheral muscular tissues or in a beating heart, almost 90% of cells are lost after 1 or 2 days [160]. Cell death limits initial retention and secondary migration. After implantation of MSCs in a decellularized tissue and its application onto the infarct area, there is a 50% secondary migration of MSCs at 8 days [161]. In non-beating peripheral muscle, after administration in mice of MSCs in collagen 1 gel, Matrigel™ or decellularized tissue (i.e., Purametrix™), there is less than 10% cellular retention of MSCs after 1 month. The same results have been found after implantation of rat cardiomyoblasts onto the MI area in a heterotopic heart transplantation model [162]. The functionalization of the scaffold with RGD may be a way of improving cellular retention. The presence of RGD in a nanofiber matrix has been shown to enhance mice bone marrow retention in ischemic limbs from 4 days to 1 month [163].

While free alginate scaffold has been found to improve remodeling after MI, this effect is lost after functionalization with the RGD peptide due to increased scaffold thickness [39,158]. The alginate functionalization with RGD induces a beneficial effect in vitro and in vivo in human endothelial cells. The functionalization of collagen with the RGD peptide, with or without cells, can be a way for providing these signals without the full protein.

## 11. Possible Use of an Empty Solid Collagen Sponge and Application to an Infarct Area without a Cellular Component, but with Growth Factors or Exosome

The collagen sponge can be functionalized with the RGD peptide and associated with a biological non-cellular agent that recapitulates most cell paracrine functionalities.

### 11.1. Possible Use of a Solid, Empty Reconstituted Collagen Scaffold Alone

Given that traditional freeze-drying processes produce scaffolds with random architecture, advances in freeze-drying technologies (DHT) offer control over ice crystal formation and segregation, enabling the development of highly ordered collagen sponges that closely imitate native supramolecular assemblies [13,78,93,118].

In humans, numerous data have also advocated the use of collagen sponges, with or without functional molecules and/or cells, in clinical (human) settings [16]. Collagen sponges have been shown to induce a substantial increase in the connective tissue thickness of palatal [164]. They have also been shown to be more effective than autologous tissues in cranial neurosurgery [165]. Collagen sponges loaded with recombinant human bone morphogenetic protein (BMP) have been used successfully in rotator cuff surgery. A collagen sponge with autologous chondrocytes has shown good short-term clinical and radiological results in large focal chondral and osteochondral defects. A collagen sponge loaded with autologous mesenchymal stem cells has also been used successfully in intervertebral disc regeneration.

Many solid collagen sponges obtained by DHT are already in clinical use, including in cardiac surgery, as hemostatic sponges. Some of these sponges have also been used in patients requiring open cardiac surgery for bypass revascularization in the context of MI for local hemostasis. All these sponges have been applied onto human heart myocardium for local hemostasis without secondary effects [16]. More recently, collagen sponges loaded with antibiotics have been used successfully in thoracic and cardiac operations [166].

In animals, DHT sponges having been applied, onto the infarct area provoked by local cryoinjury in rats. After D60, there is a total elimination of collagen sponge. A local angiogenesis in and under the patch in the infarct area has been reported [143]. There is also a trophic effect on granulose tissue. No contractile cells are observed in the patch [143]. The epicardial application of a collagen DHT patch immediately after MI has also been reported by another research group in rats up to 6 weeks, resulting also in a good integration of the scaffold, no foreign body reaction, neovascularization, and remodeling, but still with no improvement of the systolic function [167]. A DHT collagen patch, Ultrafoam^TM^, has been used to replace the full thickness of a rat right ventricle up to one month. There is a significant decrease in patch thickness. Modifications of the patch with VEGF, which also need to be covalently bound for functionality reasons [136], limited patch remodeling [136]. A collagen patch but chemically crosslinked by EDC has been implanted onto the infarct area in a rat. There are very few cells infiltrating at 3 weeks and a foreign body response is observed at the periphery [168].

### 11.2. Possible Use of a Collagen Patch with Growth Factors or Exosomes Containing Growth Factors

#### 11.2.1. Reconstituted Solid DHT Collagen Associated with Periostin

After MI, matricelluar proteins play a key role by promoting tissue sparing, tissue regeneration and angiogenesis. In many cases, the RGD functionality is involved. A collagen patch has been used for delivering the matricellular protein (i.e., periostin) that was not covalently bound and then epicardially applied 2 days after MI in a large (pig) animal model. The presence of periostin increases the ejection fraction from 31% to 41%, decreases fibrosis by 22%, and leads to a decrease in MI size at 12 weeks [169]. Integrin αv FAK/Akt activation and the RGD site of periostin contribute to the beneficial effect on the infarct area [170]. However, periostin is also associated with an increase in fibrosis of the non-infarct remote myocardium [169].

#### 11.2.2. Association of Solid Collagen Sponge “Ultrafoam™” with VEGF or Angiopoetin1

The vascular endothelial growth factor, immobilized by being covalently bound to a collagen scaffold, promotes penetration and proliferation of endothelial cells [144]. Researches have shown how the growth factor needs to be covalently bound to the scaffolds for optimal functionality [144]. In vivo, the use of collagen, with or without the VEGF, has been used to replace the full thickness of the right ventricle. VEGF-treated patches are significantly thicker than controls, and thickness correlates positively with neovascularization. Importantly, angiogenesis in VEGF scaffolds has been shown to contribute to improved cell survival and tissue formation [136].

#### 11.2.3. Association of Collagen Patch with FSLT1 (Follistatin –Like1)

A compressed collagen patch has been used in conjunction with protein FSLT1 (follistatin –like1) and applied to an infarct area 10 days after MI. FSLT1 is secreted by MSC. The application of FSTL1 via an epicardial patch stimulates the cell cycle entry and the division of pre-existing cardiomyocytes, and improves cardiac function and survival in mouse and swine models of myocardial infarction [126]. Only the application of collagen with the growth factor, and not the delivery of cells secreting the growth factor, has been shown to be effective [126]. The compressed collagen type I has a very low porosity (1 μm), which is particularly low for cellular colonization.

#### 11.2.4. Association of Collagen Patch with Exosome

As with other growth factors, collagen can be used for the local delivery of exosomes. The paracrine effect of transplanted cells is mediated in part by exosomes, nano-sized (less than 100–150 nm diameter) regulatory vesicles that are secreted by most cells and contain a variety of proteins, RNAs and growth factors. In contrast to individual bioactive components, exosomes provide a unique method for the cells to deliver a package of bioactive components. Exosomes have been shown to enhance proliferation, survival and angiogenic potential of cardiac cells in both small and large animal models. For example, exosomes of “cardiospheres” attenuate LV remodeling and improve cardiac function in swine models of acute MI [157].

## 12. Possible Use of a Solid Collagen Scaffold for Epicardial Cell Delivery in the Context of MI

In the context of MI, we have previously shown in mice [45] and humans [46,47] how the delivery of bone marrow cells, associated with a patch and epicardially applied, is superior to free intramyocardial cell injections. More recently, we have seeded human cardiosphere cell clusters in vitro in a collagen-DHT RGD scaffold or gelatin foam and have shown how the presence of RGD enhances their cardiogenic potential. With regard to cardiosphere-derived cell retention, intramyocardial injection in hyaluronic-gelatin hydrogel increases cellular retention compared to that obtained with the classical clinical intracoronary administration [73]. The effect of stem cell therapy has been found to be even better in animal models after the epicardial application of a sheet containing collagen IV and hyaluronic acid [171].

## 13. Current Challenges for Cellular Therapy after MI and the Possible Use of Collagen Scaffolds Functionalized with the RGD Peptide for the Delivery of Paracrine Human Mscs or Human Cardiopshere Cells

The clinical impact of cell-based therapy is limited by the low engraftment rate [9]. Most of the impact, including that for contractile cells, is due to the paracrine effect of transplanted cells. The delivery of an engineered cardiac tissue patch sutured onto the surface of the MI results in 10 times more engraftment than that resulting from free cell injection, even for paracrine cell delivery. The patch may provide a better environment for cell retention and survival in the context of MI. At the same time, while cardiomyocytes have a low propensity for migration outside the heart, after local administration in a patch, this is not the case for MSCs, as they already have 50% migration outside the heart after only 4 days [161]. Thus, a specific strategy should be adopted. Cases of survival, angiogenesis and electrical coupling of cellularized patches after MI have been reported after epicardial applications.

There is growing evidence that the cells should be transplanted as soon as possible after MI, probably in the first 2 days. Having a preparation of cells that is already available in the center is challenging, since cryopreservation is the classical method of MSC preservation and MSCs require 48h to recover their paracrine functionality (after DE freezing) and adhesion properties. This is still challenging in the context of MI. Immunological issues remain with hiES-CM and in relation to the scaffolds (collagen or decellularized tissue) or Matrigel™. Tissues obtained from gel have poor mechanical properties and solid scaffolds may be preferable to preserve 3D architecture, stiffness, porosity, and nutrition, and to improve the transfer and preparation of thick tissue. Endocardial cell injections are not optimal for treating the epicardial layer, where most post-MI cardiac regeneration takes place. We have shown in humans [46,47], in the context of recent MI in patients requiring sternotomy for bypass operations, and in mice [45], that the association of bone marrow cells in a collagen hemostatic patch produces better results than free cell injections for controlling ventricular remodeling.

Increasing evidence suggests that the therapeutic benefits of MSCs following transplantation can be largely attributed to the paracrine and trophic function of these cells rather than their differential potential. In addition to their well-known multi-lineage potential, an intriguing characteristic of MSCs is their ability to secrete a wide range of bioactive cytokines that can influence nearby cells via paracrine signaling such as VEGF, FGF, HGF, IGF, PDGF, ILs and MMPs. MSC paracrine functionality is controlled by indolaleamine 2,3 dioxygenase 1 (IDO). The effects of 3D biomaterials on MSC differentiation are mostly unknown, as are the effects of MSC differentiation on their functionality. The paracrine capability of MSCs decreases after differentiation towards osteocytes in a 3D collagen scaffold. At the same time, 3D culture conditions such as cluster formation (“spheroid”) increase theMSC paracrine potential. Solid 3D DHT collagen (i.e., Ultrafoam™ sponges) associated with gel of Matrigel™ enhances the paracrine function of associated human MSCs, although the effect on IDO and on cell differentiation has not been addressed. In 3D structures, MSCs are less fibrotic, secrete more cardiotrophic factors and retain anti-apoptotic and immunomodulatory functions. Among the secreted factors enhanced in the patch are BMP4, HGF, and VEGF, while the expression of PGDF remains unchanged. The expression of α-SMA was decreased in 3D collagen as compared to 2D culture on collagen. In the collagen scaffold, MSCs maintain the level of pro-inflammatory cytokines, such as IL6/IL8/Rantes, and CXCL10/IP-10, or anti-inflammatory cytokines, such as LIF, COX-2, TSG6, IDO (indolaleamine 2,3 dioxygnesase 1) and Il10. In 3D collagen scaffolds, the polarization of M2 macrophages seems to be reduced [54]. Recently, we have shown that IDO and MSC functionalities are enhanced in the 3D solid collagen scaffold compared to those obtained with 2D culture and, most importantly, our experiments have been conducted without any tumor extract such as Matrigel™ [51]. This functionality is preserved following functionalization of the scaffold with RGD and also after differentiation of MSC towards contractile myofibroblasts. The RGD is known to improve cell survival by activating Ilk and Akt. We have also shown that the RGD on a preparation of human cardiospheres containing some MSCs decreases the stress proteins [128]. Thus, the use of collagen scaffolds functionalized with the RGD peptide improves cell survival.

## 14. Future Directions and Conclusions

Cardiac tissue is a complex 3D environment where the different cells (i.e., mainly cardiomyocytes, MSCs, fibroblasts/myofibroblasts and endothelial cells) interact with structural collagen type I and type III networks, fibronectins, laminin and “matricilin proteins” [23,172]. Integrins recognizing the RGD motif play a key role during cardiac development [18], pressure overload [19] and after MI [20,21,22,23]. Most cells interact with their environment with integrin mechanoreceptors recognizing oligopeptides on 3 main types of proteins: collagen, laminin and the RGD peptide present on fibronectin and vitronectin proteins [12,23,62,173].

In collagen, in 2D or 3D environments, researches have revealed very early spontaneous synthesis of laminin by cardiomyocytes that governs the organization of the contractile apparatus [17]. On collagen, the RGD peptide is present but non-functional [14]. Cardiomyocytes seeded in a collagen scaffold without functionalization with the RGD do not have all the signaling molecules necessary for their optimal differentiation. Thus, we propose functionalizing the collagen with the RGD peptide to provide these additional signals [61].

Thanks to the use of a solid DHT collagen scaffold, composed of collagen type I (50%) and collagen type III (50%) collagen and of low stiffness (around 1 kPa) [128] and high porosity [128], and after functionalization with the RGD peptide, we have been able to develop a very efficient contractile tissue in the presence of neonatal rat cardiomyocytes [48,51,52,53]. Moreover, this was achieved without the need for the classical tumor extract, such as Matrigel™ and needs for bioreactors, as previously reported in studies using the same solid DHT collagen scaffold [41,42,43].

With the same scaffolds, we also reported how functionalization with the RGD promotes the differentiation of human MSCs [51,52,53] or “human Cardiospheres” [128] towards a contractile phenotype. Interestingly, in these 3D scaffolds, even after differentiation towards contractile myofibroblasts, the hMSC paracrine function is enhanced as compared to classical 2D cultures [51,54].

Gelatin is the heat-denaturized form of collagen and, unlike collagen, contains a functional RGD peptide. For cells, including cardiac heart cells, the pull on RGDs covalently bound to undenatured collagen is not the same as the pull on RGDs present on gelatin, which is a denatured protein [147]. Functionalization of collagen with the RGD peptide [48] can potentially improve most cardiac cell therapies.

Optimization of the recognition of key integrins may be a way for the control of cell retention and differentiation as well as a means of enhancing cell survival in a hypoxic and damaged myocardium and of decreasing secondary migration [34]. Application of stem cells as an epicardial patch after MI may be also a way for controlling cells’ microenvironment and preserve their immune functionality after implantation [54,174,175,176], and thereby possibly a new approach for improving the results of cell therapy after MI.

More generally, functionalization of the collagen polymers with the RGD may be a way for improving contractility in collagen-based constructs. With regard to contractile patches, the epicardial application of a true contractile patch can be a way to physically remodel the ventricles after MI.

## Data Availability

Not applicable.
